# Structural informatics approach for designing an epitope-based vaccine against the brain-eating *Naegleria fowleri*

**DOI:** 10.3389/fimmu.2023.1284621

**Published:** 2023-10-30

**Authors:** Asifa Sarfraz, Tehreem Ul Wara, Sheheryar, Ke Chen, Shahid Habib Ansari, Aqal Zaman, Umar Nishan, Anwar Iqbal, Riaz Ullah, Essam A. Ali, Mohibullah Shah, Suvash Chandra Ojha

**Affiliations:** ^1^ Department of Biochemistry, Bahauddin Zakariya University, Multan, Pakistan; ^2^ Department of Biochemistry and Molecular Biology, Federal University of Ceara, Fortaleza, Brazil; ^3^ Department of Infectious Diseases, The Affiliated Hospital of Southwest Medical University, Luzhou, China; ^4^ Department of Microbiology & Molecular Genetics, Bahauddin Zakariya University, Multan, Pakistan; ^5^ Department of Chemistry, Kohat University of Science & Technology, Kohat, Pakistan; ^6^ Department of Chemical Sciences, University of Lakki Marwat, Khyber Pakhtunkhwa, Pakistan; ^7^ Department of Pharmacognosy, College of Pharmacy, King Saud University, Riyadh, Saudi Arabia; ^8^ Department of Pharmaceutical Chemistry, College of Pharmacy, King Saud University, Riyadh, Saudi Arabia

**Keywords:** primary amoebic meningoencephalitis, parasite, epitope, brain-eating, *Naegleria*, immunoinformatics, reverse vaccinology

## Abstract

Primary Amoebic Meningoencephalitis (PAM), a severe lethal brain disease, is caused by a parasite, *Naegleria fowleri*, also known as the “brain-eating amoeba”. The chances of a patient’s recovery after being affected by this parasite are very low. Only 5% of people are known to survive this life-threatening infection. Despite the fact that *N. fowleri* causes a severe, fatal infection, there is no proper treatment available to prevent or cure it. In this context, it is necessary to formulate a potential vaccine that could be able to combat *N. fowleri* infection. The current study aimed at developing a multi-epitope subunit vaccine against *N. fowleri* by utilizing immunoinformatics techniques and reverse vaccinology approaches. The T- and B-cell epitopes were predicted by various tools. In order to choose epitopes with the ability to trigger both T- and B-cell-mediated immune responses, the epitopes were put through a screening pipeline including toxicity, antigenicity, cytokine-inductivity, and allergenicity analysis. Three vaccine constructs were designed from the generated epitopes linked with linkers and adjuvants. The modeled vaccines were docked with the immune receptors, where vaccine-1 showed the highest binding affinity. Binding affinity and stability of the docked complex were confirmed through normal mode analysis and molecular dynamic simulations. Immune simulations developed the immune profile, and *in silico* cloning affirmed the expression probability of the vaccine construct in *Escherichia coli* (*E. coli)* strain K12. This study demonstrates an innovative preventative strategy for the brain-eating amoeba by developing a potential vaccine through immunoinformatics and reverse vaccinology approaches. This study has great preventive potential for Primary Amoebic Meningoencephalitis, and further research is required to assess the efficacy of the designed vaccine.

## Introduction

*Naegleria fowleri*, also recognized as the “brain-eating amoeba”, is a eukaryotic, highly pathogenic, and free-living amoeba. Natural habitats for *N. fowleri* include soil and freshwater, and warm water temperatures are assumed to contribute to its occurrence. It belongs to the phylum Percolozoa, class Heterolobosea, and is the etiological agent of Primary Amoebic Meningoencephalitis (PAM) (1, 2). In this disease, the brain becomes infected when free-living amoebae enter through the nose from freshwater. *N. fowleri* trophozoites can enter the nose, break through the nasal mucosa, and then move through the olfactory neurons to the central nervous system (CNS) (3). It is known that these amoebae release soluble substances such as proteases, acid hydrolases, and secreted phospholipases to name a few, that cause the death of nerve cells (4). PAM has been linked to contact with *N. fowleri* containing water. The amount of time that elapses between exposure to the organism and the appearance of clinical symptoms, including rhinitis, fever, and headache, can range from 2 to 3 days and remain for 7 to 15 days, ultimately causing the death of the patient (5). The earliest signs and symptoms are a bi-frontal headache, fever, or convulsions that can progress to paralysis, hallucinations, or a late-stage coma within the first nine days following water exposure (6).

PAM is a rarely occurring disease but is highly fatal, with a fatality rate of 95-97% (2). Just 5% of people are known to have survived this deadly infection after being affected (7). It has been reported that there are roughly 380 fatalities each year caused by *N. fowleri* worldwide, or 16 fatalities on average in the US alone (8). The PAM is probably ubiquitous, especially in developing nations with erratic medical reporting and warm climates. The threat posed by *N. fowleri* may increase as a result of climate change, which has been linked to an increase in aquatic recreational activities (9), freshwater temperatures, and extreme weather events. On the basis of an increase in case reports over the past ten years, *N. fowleri* has lately been recommended as an emerging pathogen (10).

To date, there is no effective treatment in the form of drugs or vaccines available against this parasite. A somewhat effective drug against PAM is amphotericin B, which has shown good results in some of the patients affected by this parasite (11); however, at the same time, it shows some serious side effects (12). Moreover, miltefosine and other antimicrobials are becoming more widely used for successful treatment, but this depends on early diagnosis, which is difficult due to the relative rarity of amoebic meningitis (13). As a result, there is a need for parasite preventive measures and/or control regulations in water bodies, which are tragically only implemented in a few nations at the moment (14).

Vaccination is always considered a key therapeutic mechanism against different pathogens. Previous studies oriented towards the development of a vaccine against *N. fowleri* demonstrated a potential therapeutic strategy. During such attempts, immunization of experimental mice with live or dead *N. fowleri* (15) or with the cultural medium (16) where the parasite was grown provided significant protection. Similarly, the use of Cry1Ac protoxin from Bacillus thuringiensis co-administered with parasite lysate also provided significant results in the experimental animals (17). Further studies indicated that *N. fowleri* activates the Neutrophil Extracellular Trap (NET) and releases distinctive components, but trophozoites usually manage to escape from this trap. Polymorphonuclear cells (PMNs) and NET production are highlighted by the fact that trophozoites are vulnerable to neutrophil activity after being opsonized with human IgG (18). In another study, new antigenic compounds, such as the nfa1 gene, that reacted with both infected and immune sera were discovered using immuno-screening. The pseudopodia-located Nfa1 protein may have an impact on the movement of amoeba (19). The highly pathogenic amoeba exhibits quicker movement. Therefore, vaccination with recombinant Nfa1 elicits a potent immunological response, including particular IgG and IgA antibodies, resulting in 100% survival. Furthermore, the Nfa1 gene was inoculated in mice affected by PAM, and it was observed that a 90% survival rate was observed in those mice (19).

Although these are some interesting possibilities, the actual utility of these vaccinations has not yet been established, despite their potential importance in preventing a potential spike in *N. fowleri* infections. Further investigation is necessary, and if effective, vaccination could considerably help areas where PAM is a serious public health issue. Therefore, our study employed reverse vaccinology techniques to develop different peptide vaccine constructs against the *N. fowleri* parasite by utilizing T and B-cell epitopes, adjuvants with high immunogenicity, and linkers. The designed constructs had a strong immune profile and were found to have a very minimal chance of causing toxicity or allergenicity. *In silico* cloning of the vaccine showed its efficacy in translation and expression in *Escherichia coli* (*E. coli*) strain K12.

## Materials and methods

**Figure 1:**
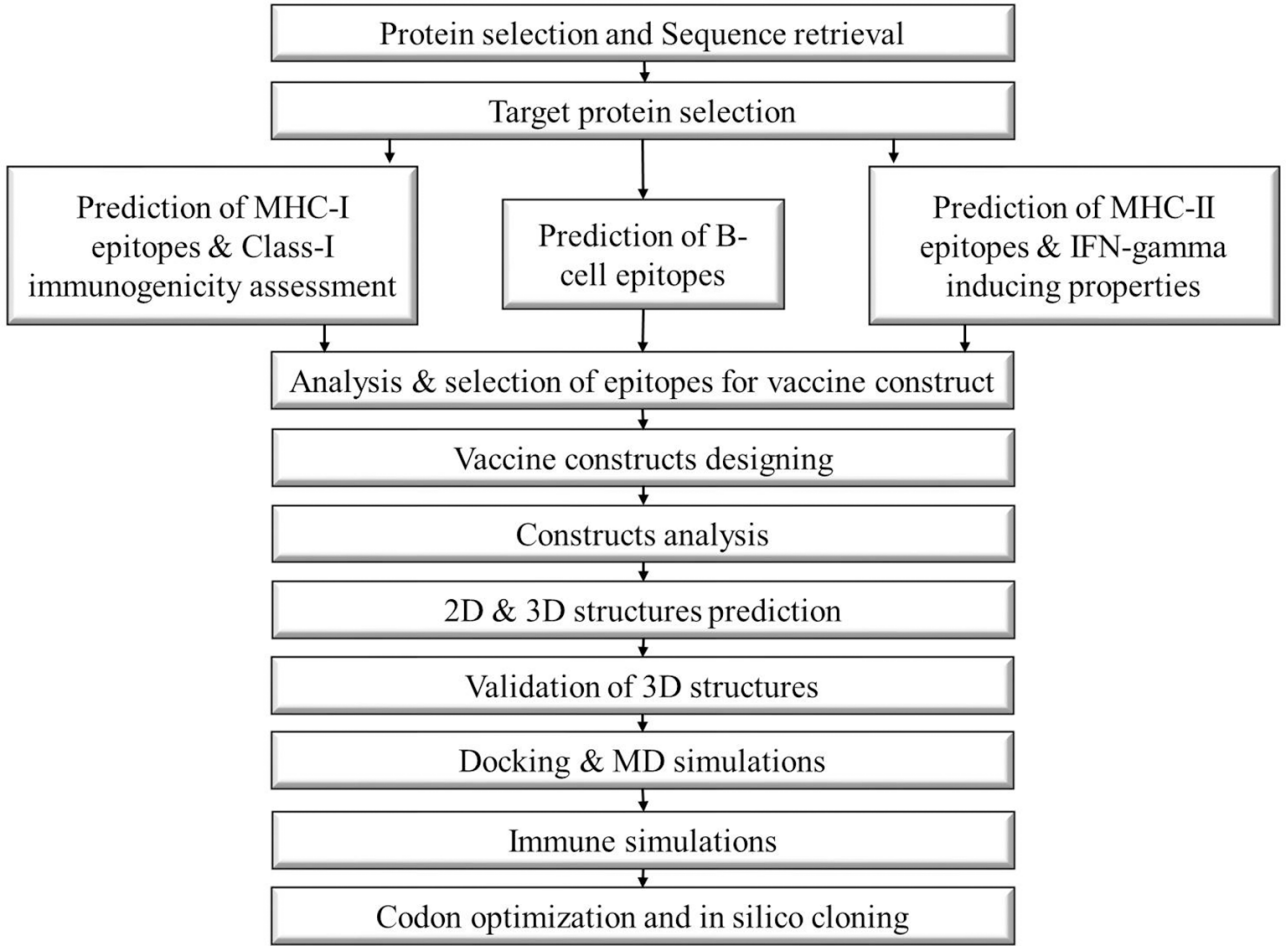
Schematic diagram of the steps followed to construct a multi-epitope vaccine against Naegleria fowleri.

The steps involved in the formation of vaccine constructs against *N. fowleri* are depicted in the schematic diagram below (**Figure 1**).

### Protein sequence retrieval and selection of candidate proteins

After an in-depth literature survey, six proteins (P42661, Q6B3P1, Q9NH76, Q95UJ2, A0A6A5CB91, P27131) of *N. fowleri* were identified that can be used as vaccine candidates. Their sequences were downloaded from the UniProtKB database (https://www.uniprot.org/). The antigenic potential of these proteins was identified via the Vaxijen v2.0 server (20) while their allergenic profile was determined using the AllergenFP server (21). Furthermore, their physicochemical properties were checked using the Protparam Expasy server (22) and the TMHMM server was used to check the topology values of all proteins. The most antigenic, stable, and non-allergenic proteins were selected as the vaccine candidates (23, 24).

### T-cell epitopes prediction

The Immune Epitope Database (IEDB) (25), an open-access and user-friendly database, was used for the determination of T-cell epitopes, which participate in immune responses (26). For MHC-I or CTL epitope prediction, artificial neural networks (ANN) were applied using the NetMHCpan EL 4.1 method technique. The protein sequences were used as the input, all the parameters were kept as default, and the job was submitted. Furthermore, for MHC-II or HTL epitope prediction, the IEDB-recommended 2.22 method was used with default parameters; protein sequences were provided as the input, a full HLA reference allele set was used so that the resulting epitopes would cover the HLA present in the whole world, and the length of the predicted epitopes was set to 15-mer (27). The ranking of the resulting epitopes was done on the basis of their percentile ranks. The smaller the rank, the higher the affinity of epitopes to bind with MHC-I or MHC-II cells.

### B-cell specific epitopes prediction

B-cell or LBL epitopes perform crucial functions in immunological reactions mediated by antibodies during the development of multi-epitope vaccines. Bepipred Linear Epitope Prediction 2.0, present within the IEDB-AR (28), was formed on the basis of a random forest algorithm that uses epitopes as its training data. This tool is the most effective and accurate in predicting linear B-cell epitopes. In this study, the Bepipred Linear Epitope prediction tool was used for the determination of linear B-cell epitopes. The preferred length of the selected epitopes was between 10 and 40.

### Analysis of CTL, HTL, and LBL epitopes

The antigenicity and allergenicity of the epitopes were determined using Vaxijen v2.0 and the AllergenFp server. Both of these servers are alignment-independent servers because they utilize the autocross-covariance (ACC) technique. The accuracy of Vaxijen v2.0 ranges from 70-89% and that of the AllergenFp server is 0.879. Thus, these are reliable tools for antigenicity and allergenicity prediction, respectively (20, 21). The Toxinpred server was used to check the toxicity of the epitopes, which is a unique *in silico* technique for toxicity prediction (29). To be a vaccine candidate, the selected proteins must have the ability to elicit an immune response. Thus, the IFN-gamma, Il4, and IL10-inducing properties of the epitopes were determined using IFN-epitope, IL4-pred, and IL10-pred servers, respectively.

### Multi-subunit vaccine construction and analysis

Adjuvants, linkers, and epitopes are the necessary elements in constructing a vaccine. Adjuvants are required to reduce the number of injections and antigen content and induce potent immune responses (30). Linkers play a crucial role in joining the epitopes and assisting in the proper functioning of vaccines (31). Epitopes are antigenic, non-toxic, and non-allergenic components recognized by the body’s defense system and that initiate the immune response (32). The selected epitopes were linked using linkers, and adjuvants were added to boost the immunogenicity. MHC-I epitopes are generally joined using an AAY linker, but in this study, only one epitope of MHC-I was selected, so this linker was not used. The MHC-II epitopes were linked using a GPGPG linker, and the B-cell epitopes were linked with the KK linker. Three different adjuvants L7/L12 Ribosomal-protein adjuvant, Beta-defensin adjuvant, and Granulocyte-macrophage colony adjuvant were used to make three different vaccine constructs.

### Immunoinformatic evaluation of construct

VaxiJen v2.0, AntigenPro, Protein-sol, Solpro, AllergenFP, and the Protparam Expasy servers were used to evaluate the physicochemical properties of the vaccine construct. Using these tools, users can obtain in-depth information on the various characteristics, such as the antigenicity, via the VaxiJen v2.0 and AntigenPro servers to access the vaccine’s immunogenicity (33, 34). Both approaches are alignment-independent; however, VaxiJen 2.0 bases its operation on a variety of physicochemical properties of the protein (20), while AntigenPro is a server based on microarray analysis data through machine learning algorithms and has an accuracy rate of 82% (35). The preferred antigenicity score of Vaxijen v2.0 and AntigenPro is >0.75 and >90%, respectively. Allergenicity was determined through the AllergenFP server, which distinguishes between allergens and non-allergens based on their physicochemical and structural characteristics (21). Allergenicity was predicted to ensure that the vaccine doesn’t cause any allergic reactions after entering the body. Solubility was determined via Solpro and Protein-Sol Sol servers. Solpro is a server based on sequence prediction by utilizing the two-tier SVM approach and has an accuracy rate of 74.15% (36). Protein-sol is a server for the prediction of protein solubility based on the observation of a bimodal distribution of protein solubilities for *E. coli* proteins (37). Solubility guarantees consistent dissolution of essential components for precise dose and vaccine distribution. The ProtParam Expasy server predicts various parameters such as the GRAVY value, aliphatic index, instability index, molecular weight, and the theoretical Pi (38). The GRAVY value reports about the polarity of the vaccine; the aliphatic index ensures the thermostability; the instability index tells whether the vaccine is stable or not; the molecular weight affects the antigenicity of the vaccine, and the theoretical Pi shows the hydrophobic and hydrophilic nature of a vaccine.

### Secondary and tertiary structure prediction

The 2D structure of the constructs was formed by the PSIPRED web server. PSIPRED is a highly accurate tool for 2D structures and includes the two feed forward neural networks that analyze the results obtained from PSI-BLAST (39). The tertiary structure was formed using the SWISS-MODEL, which is one of the most popular structure modeling servers (40). Visualization of the predicted structures was done with PyMOL and the Discovery Studio 3.5 programs.

### Refinement and validation of tertiary structure

The quality of the tertiary structures predicted by SWISS-MODEL was improved by refining. The GalaxyRefine is a server for the refinement of the protein model structure that excels at enhancing local structure quality. It displays a generally modest improvement in the structural quality of the backbone. GalaxyRefine server enhances the input models with a probability of >50% (41). Whether the protein structure models are determined computationally or experimentally, the Ramachandran plot provides a validation check of the input structures. For a better protein structure, the number of residues in the favored regions must be >90% (42). Furthermore, the ERRAT plot is also a verification algorithm for the protein structures, which represent the non-bonded interactions. It is particularly useful for monitoring the development of crystallographic model construction and refinement. The preferred ERRAT quality factor value is ≥0.5%; the higher the score, the higher the quality of the proteins. Therefore, the refined structures were validated by the Ramachandran plot, which was formed using the Vadar server (43) and the ERRAT quality factor value predicted by the SAVES server (44).

### Conformational B-cell epitopes prediction

Conformational B-cell epitopes are surface-exposed and easily accessible to the solvent. The Ellipro server is very beneficial for the prediction of the residues of vaccines that are involved in the formation of conformational B-cell epitopes. ElliPro predicts conformational epitopes by combining geometric characteristics with an antigen’s predilection for a single amino acid (45).

### Molecular docking and normal mode analysis

The molecular docking of the vaccines was performed with the human immune receptors, TLR2 (PDB ID: 2Z7X) and TLR4 (PDB ID: 3FXI) to identify their binding affinities. The preparation of vaccines and receptors was done using the MOE software. After then, both the receptor (TLR2 and TLR4) and the ligand (Vaccines) prepared structures in PDB format were uploaded to the ClusPro2.0 server, which is a protein-protein docking tool that employs the Fourier correlation algorithm and filters out models with a combination of desolvation and electrostatic energies (46). The best docked vaccine-receptor complexes were then subjected to normal mode analysis by the IMODs server in order to check the stability of the complexes (47).

### MD simulation

The V1-Receptor complexes were subjected to Molecular Dynamic Simulations for determining the complex stability. The TLR2 & TLR4 as receptors and the vaccine V1 as ligand were prepared as per the standard protocol of GROMACS as performed in our recent study (23). The constructed complexes were subjected to production run of 100 ns and results were analyzed using vmd and in house scripts.

### Codon optimization and *in-silico* cloning

The vaccine sequence of the best complex was back translated, and then codon optimization was done to enhance the codon usage of the recombinant genes while preserving the input amino acid sequences. This was done via the Java Codon Adaptation Tool (JCAT) (48) with some specific settings, i.e., the *E. coli* strain K12 was selected as the host organism, all the additional settings were selected to avoid rho-independent transcription terminators, cleavage sites of restriction enzymes, and prokaryotic ribosome binding sites, and to allow only partial optimization in order to apply site directed mutagenesis during the run. The results of the optimization were analyzed by the GC content and the CAI value, which are indicators of gene expression levels, as well as codon usage bias markers. Overall, codon optimization was done to maximize efficiency and yield while keeping the correct amino acid sequence in order to optimize the creation of the vaccination protein in the host organism. After that, the SnapGene software was used for the cloning of the optimized codon sequence (49). An pET-28a(+) plasmid vector containing the designed vaccine gene was inserted for expression in the host. The pET-28a(+) plasmid was selected because it has been extensively used in expression studies (50).

### Immune simulations

The C-ImmSim server was used to elucidate the sufficient immune response of our best docked vaccine-receptor complex. It uses machine-learning techniques and a position-specific score matrix to predict the immunogenicity of the vaccines. Three unique anatomical locations: the thymus, lymph node, and bone marrow, which are essential for healthy immune system function, were simulated to create this server (51). The vaccine sequence was provided as input and three injections (1, 84 and 170) were administered to check the immune response.

## Results

### Protein sequence retrieval and analysis

Six proteins namely Nf314 (P42661), HSP70 (Q6B3P1), Nfa1(Q9NH76), Mp2CL5 (Q95UJ2), Nf23 (A0A6A5CB91), Actin-1 (P27131) involved in the pathogenicity of *N. fowleri* were identified from the literature, and their sequences were retrieved from NCBI and UniProt. These proteins were analyzed to check their potential to be used as vaccine targets. The parameters checked were the GRAVY value, Aliphatic index, instability index, topology value, allergenicity, and antigenicity. The preferred values of topology were 0 or 1, while the GRAVY value should be negative. The vaccine target must also be stable. After the complete analysis, only two proteins, namely, heat shock protein Hsp70 (ID: Q6B3P1) and nf23 (ID: A0A6A5CB91), were found to have the potential to be candidate proteins (**Table 1**) and were used for downstream analysis.

**Table 1:** Physicochemical, antigenicity and allergenicity analysis of target proteins.

Sr. No.	Protein ID	Name	No. of residues	Mol. weight	GRAVY value	Theoretical Pi	Aliphatic index	Instability index	Topology value	Antigenicity	Allergenicity
1	AAA29384.1	Nf314	482	53848.03	-0.297	7.95	74.83	37.61 (stable)	0	Non-antigen	Allergen
**2**	AAT92549.1	**Hsp70**	**659**	**71407.6**	**-0.444**	**5.14**	**75.22**	**34.02 (stable)**	**0**	**Antigen**	**Non-allergen**
3	AAF35899.1	Nfa1	119	13403.22	-0.269	5.91	83.61	37.8 (stable)	0	Non-antigen	Non-allergen
4	AAL09154.1	Mp2CL5	181	19932.22	-0.338	6.82	72.6	22.61 (stable)	0	Non-antigen	Non-allergen
**5**	A0A6A5CB91	**Nf23**	**229**	**25558.28**	**-0.628**	**9.04**	**61.22**	**33.03 (stable)**	**0**	**Antigen**	**Non-allergen**
6	P27131	Actin-1	375	41727.88	-0.164	5.26	85.28	29.96 (stable)	0	Non-antigen	Non-allergen

The bold represent the proteins selected for the vaccine designing.

### Prediction and evaluation of T-cell epitopes

T-cells consist of two types of immune cells, namely, MHC-I and MHC-II cells, and hence the epitopes of these cells were predicted.

### MHC-I and MHC-II epitopes identification

The IEDB recommended 2.22 MHC-I prediction resulted in 35128 epitopes for Hsp70 and 11908 for Nf23, respectively. Out of these, 89 unique epitopes in the case of Hsp70 and 47 for Nf23 with ranks 0.2 and 0.3, respectively, were identified. Out of these, the top 10 epitopes of both proteins were selected for further analysis, and finally, only one MHC-I epitope showing the best antigenicity, non-allergenicity, and immunogenicity was selected for vaccine design (**Supplementary Tables 1**, **2**).

The MHC-II prediction resulted in 17416 epitopes for Hsp70 and 5806 for Nf23, respectively. Among the 17416 epitopes of Hsp70, 134 unique epitopes with percentile rank 1 were scaled. Out of these, the top 10 were used for further analysis, and the four most antigenic and non-allergenic peptides were chosen for vaccine construction. While, among the 5806 epitopes of Nf23, 70 unique values having the percentile rank 3 were determined. Out of these, 9 epitopes with the lowest rank were further analyzed, and finally, two of their epitopes showed the potential to be vaccine targets after the antigenicity and allergenicity analyses. Furthermore, the IFN-gamma, IL4, and IL10-inducing properties of MHC-II binding epitopes were also determined (**Supplementary Tables 3**, **4**).

### B-cell epitopes prediction and evaluation

The BepiPred Linear Epitope prediction server was employed for the determination of B-cell epitopes. This resulted in 10 epitopes for Hsp70 and 4 for Nf23, with lengths ranging from 10 to 40. Their toxicity, allergenicity, and antigenicity were determined, and finally, 7 epitopes in the case of Hsp70 and 1 in the case of Nf23 showing the best results were selected for vaccine construction (**Supplementary Tables 5**, **6**).

### Designing of multi-epitope construct

**Figure 2:**
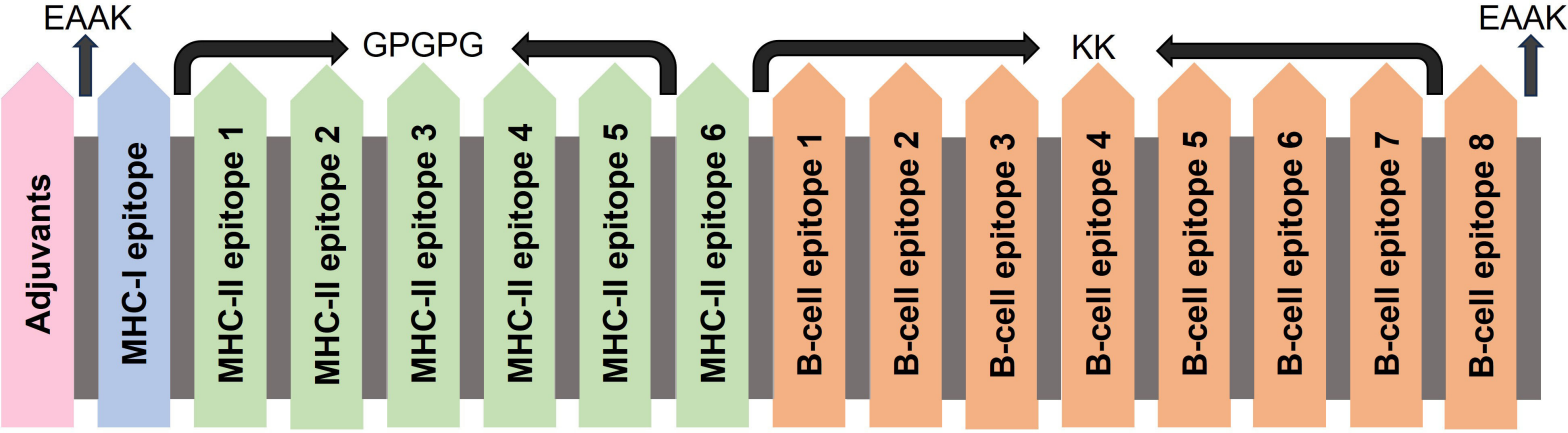
Construction of multi-epitope vaccine by using linkers and adjuvants.

Constructs are designed by joining the epitopes. For this purpose, different linkers (AAY for MHC-I, GPGPG for MHC-II, and KK for B-cells) are used. These are flexible and provide flexibility to the construct. In our study, only 1 MHC-I molecule showed the potential to become the vaccine construct, so the AAY linker was not used here. Both the other linkers were the same. An EAAK linker was also added at the start and end of the construct to provide rigidity at the ends. Additionally, adjuvants play an effective role in enhancing the immune profiles of vaccine constructs. We used three different types of adjuvants to construct three vaccines (**Table 2**). These three adjuvants were the L7/L12 Ribosomal-protein adjuvant, Beta-defensin adjuvant, and Granulocyte-macrophage colony adjuvant (**Figure 2**).

**Table 2:** Vaccine constructs formation using different adjuvants.

Vaccine	Adjuvant	Vaccine construct
**V1**	L7/L12 Ribosomal-protein adjuvant	MSDLKNLAETLVNLTVKDVNELAAILKDEYGIEPAAAAVVMAGPGAEAAEEKTEFDVILKSAGASKLAVVKLVKDLTGAGLKEAKDMVDGAPAAIKSGISKDEAEALKKQLEEAGAEVELKEAAKSQIDDVVLVGPGPGEEERLIGDAAKNQVAGPGPGIEVTFEIDANGIMKVGPGPGHWPFKVITKGDDKPYGPGPGEDPNLAGKISDADKNGPGPGASTPSSFVLHNQQDGGPGPGMNFYSKSPKTQSSQHKKKDFFNGKELCKSINKKGKETRVLLIDVTPLSLGIETAGGKKERNTTIPCKKSKKEGERTMTKDNHLKKTITNDKGGLSKEEIEEMLKQAEQMKSQDDELRKEVEAKNHKKVEDPNLAGKISDADKNTIKKKKQGAGGAGAGAGGFPGAGAGGFPGAGGFPGAGAGAGGESSTGGAQPKFEDKKPFGGYQEKSGKEAAK
**V2**	Beta-defensin adjuvant	GIINTLQKYYCRVRGGRCAVLSCLPKEEQIGKCSTRGRKCCRRKKEAAKSQIDDVVLVGPGPGEEERLIGDAAKNQVAGPGPGIEVTFEIDANGIMKVGPGPGHWPFKVITKGDDKPYGPGPGEDPNLAGKISDADKNGPGPGASTPSSFVLHNQQDGGPGPGMNFYSKSPKTQSSQHKKKDFFNGKELCKSINKKGKETRVLLIDVTPLSLGIETAGGKKERNTTIPCKKSKKEGERTMTKDNHLKKTITNDKGGLSKEEIEEMLKQAEQMKSQDDELRKEVEAKNHKKVEDPNLAGKISDADKNTIKKKKQGAGGAGAGAGGFPGAGAGGFPGAGGFPGAGAGAGGESSTGGAQPKFEDKKPFGGYQEKSGKEAAK
**V3**	Granulocyte-macrophage colony	MWLQSLLLLGTVACSISAPARSPSPSTQPWEHVNAIQEARRLLNLSRDTAAEMNETVEVISEMFDQEPTCLQTRLELYKQGLRGSLTKLKGPLTMMASHYKQHCPPTPETSCATQIITFESFKENLKDFLLVIPFDCWEPVQEEAAKSQIDDVVLVGPGPGEEERLIGDAAKNQVAGPGPGIEVTFEIDANGIMKVGPGPGHWPFKVITKGDDKPYGPGPGEDPNLAGKISDADKNGPGPGASTPSSFVLHNQQDGGPGPGMNFYSKSPKTQSSQHKKKDFFNGKELCKSINKKGKETRVLLIDVTPLSLGIETAGGKKERNTTIPCKKSKKEGERTMTKDNHLKKTITNDKGGLSKEEIEEMLKQAEQMKSQDDELRKEVEAKNHKKVEDPNLAGKISDADKNTIKKKKQGAGGAGAGAGGFPGAGAGGFPGAGGFPGAGAGAGGESSTGGAQPKFEDKKPFGGYQEKSGKEAAK

### Evaluation of constructs

The allergenic and antigenic profiles of constructs were checked by AllergenFp, Vaxijen v2.0, and AntigenPro servers. It identified that all three constructs were highly antigenic and non-allergenic. Solubility was determined by Protein-sol and the SolPro servers, which indicated that the constructs are soluble. The physicochemical properties analysis predicted that our constructs are stable and polar, and two are basic while one is slightly acidic in nature (**Table 3**).

**Table 3:** Physicochemical, antigenicity and allergenicity analysis of the designed vaccine constructs.

Parameters	V1		V2		V3	
	Measurement	Indication	Measurement	Indication	Measurement	Indication
Molecular weight	47031.06	Appropriate	39699.89	Appropriate	50720.3	Appropriate
No. of Amino acids	454	Appropriate	378	Appropriate	476	Appropriate
Nucleotides	1362	Appropriate	1134	Appropriate	1428	Appropriate
Solubility by Prot-Sol	0.657	Soluble	0.769	Soluble	0.594	Soluble
Solubility by Sol-pro	0.661	Soluble	0.739741	Soluble	0.607273	Soluble
Aliphatic index	66.94	Thermostable	54.26	Thermostable	61.95	Thermostable
Instability index	25.78	Stable	29.88	Stable	39.5	Stable
Antigenicity by Vaxijen	0.7784	Antigenic	0.9064	Antigenic	0.8589	Antigenic
Antigenicity by AntigenPro	0.91353	Antigenic	0.930108	Antigenic	0.961066	Antigenic
Theoretical Pi	6.34	Almost neutral	9.34	Basic	7.56	Basic
Allergenicity	Non-allergen	Non-allergenic	Non-allergen	Non-allergenic	Non-allergen	Non-allergenic
Grand value of hydropathicity (GRAVY)	-0.658	Hydrophilic	-0.879	Hydrophilic	-0.705	Hydrophilic

### Prediction of 2D and 3D structures

The secondary structures were generated using the Psipred server (**Supplementary Figure 1**). The obtained 2D structures contained 47.14% alpha helix, 9.69% extended strand, and 43.14% random coil in case of V1, 40.48% alpha helix, 13.49% extended strand, and 46.03% random coil in case of V2, and 36.55% alpha helix, 11.13% extended strand, and 52.31% random coil in case of V3. Furthermore, the hydrophobicity and hydrophilicity of the vaccines were also determined using the Psipred server (**Supplementary Figure 2**).

**Figure 3:**
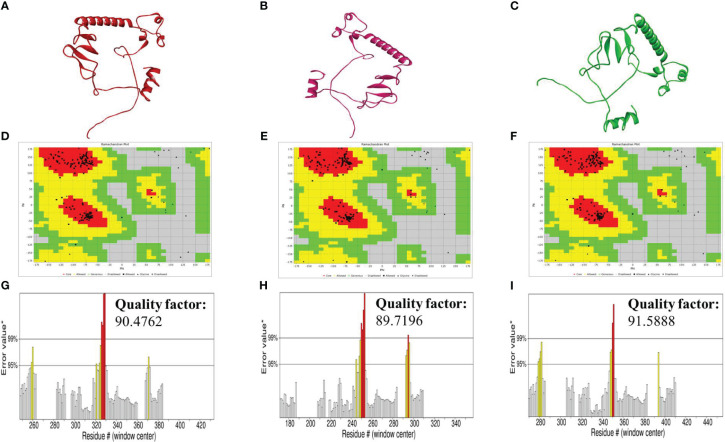
**(A–C)** Tertiary structures of the vaccines V1 (red), V2 (hot-pink), and V3 (green) generated by the SWISS-model server **(D–F)** Ramachandran plot of vaccines V1, V2, and V3 formed by Vadar server **(G–I)** ERRAT graphs plot of vaccines V1, V2, and V3 indicating the quality factor of the vaccines formed by the SAVES server.

The tertiary structures of the constructs were built using the Swiss model server. The resulting models were refined using the GalaxyRefine server (**Figures 3A–C**). After then, the quality of the refined structures was determined by the ERRAT plot using the SAVES server and the Ramachandran plot using the Vadar server. The Ramachandran plot identified by the vaccines validated their 3D structures, as most of the residues of our constructs were in the most favored regions. The amino acid residues of the vaccines in the most favored regions were 94.2% in the case of V1 and V2, and 96.1% in the case of V3. This indicated that all our constructs are of good quality (**Figures 3D–F**). The ERRAT quality factor values were found to be 90.4762, 89.7196, and 91.5888 for V1, V2, and V3, respectively, as indicated in the form of ERRAT plots (**Figures 3G–I**).

### Conformational B-cell epitopes prediction

The B-cell epitopes are involved in the production of antibodies and cytokinin to produce the immune response. The vaccine’s residues involved in the formation of conformational B-cell epitopes were identified using the Ellipro server available within the IEDB analysis resource. The residues that scored the best in the formation of the conformational B-cell epitopes are indicated in **Supplementary Table 7** and **Supplementary Figure 3**.

### Molecular docking and normal mode analysis

**Figure 4:**
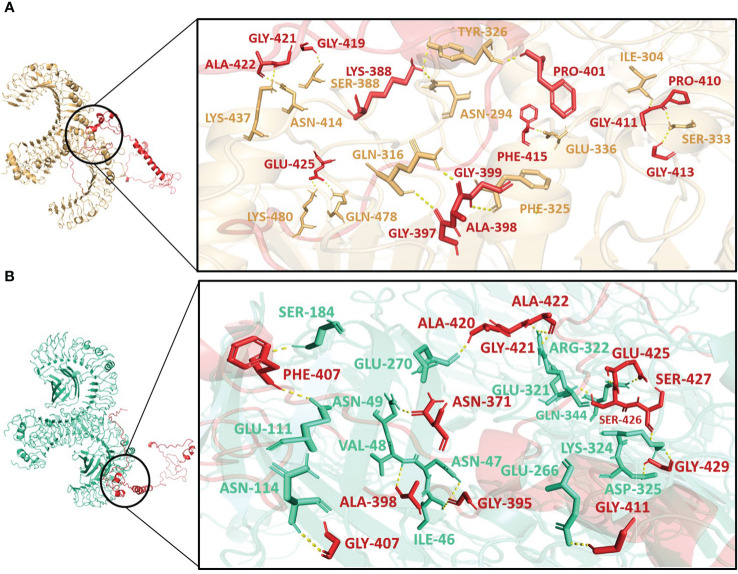
Interactions of the vaccine V1 (red) with **(A)** TLR2 (light-orange) and **(B)** TLR4 (green-cyan).

**Figure 5:**
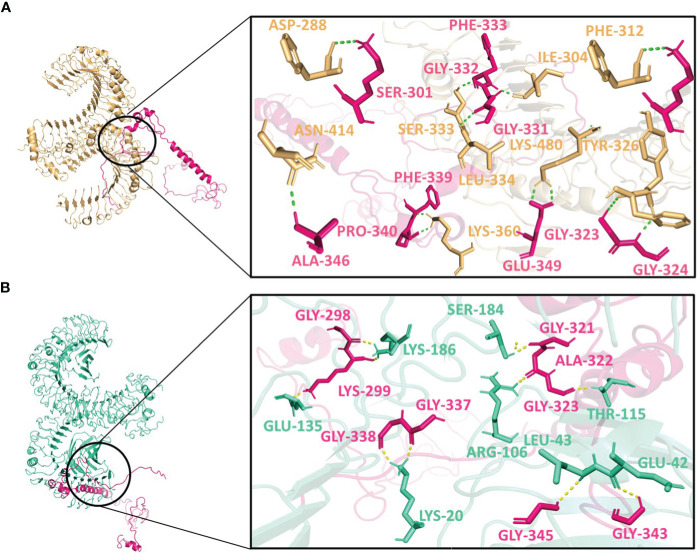
Interactions of the vaccine V2 (hot-pink) with **(A)** TLR2 (light-orange) and **(B)** TLR4 (green-cyan).

**Figure 6:**
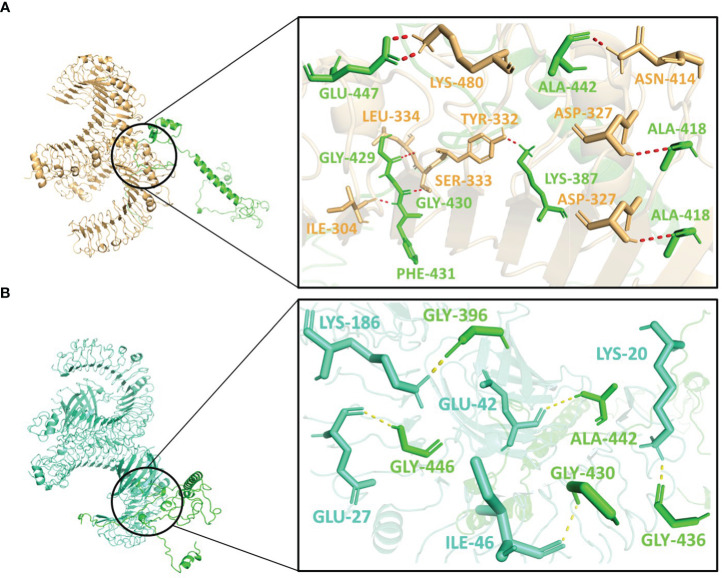
Interactions of the vaccine V3 (green) with **(A)** TLR2 (light-orange) and **(B)** TLR4 (green-cyan).

Molecular docking was performed using the ClusPro server 2.0. The resulting vaccine-receptor complexes were then analyzed, and their docking scores were determined. The interactions of vaccine constructs (V1, V2, and V3) and Toll-like Receptors (TLR) 2 and 4 were determined which provided valuable insights on potential implications for vaccine design and immune response modulation (**Figures 4**–**6**). When analyzing the interactions of V1 with TLR2 and TLR4, we observed notable interactions with specific residues (**Supplementary Table 8**). With TLR2, V1 depicted interactions with 13 residues, forming a substantial 15 hydrogen bonds with the lowest energy of -1027.0 kJ/mol (**Figure 4A**), and with TLR4 it also involved 13 residues, forming a network of 18 hydrogen bonds, with the lowest energy of -1397.6 kJ/mol (**Figure 4B**). It mostly involved glycine residues in the case of both receptors. These interactions indicate the robust binding of V1 with TLR2 and TLR4, possibly contributing to a complex immune response activation mechanism.

V2 depicted the lowest energy of -1089.3 kJ/mol with TLR2 and -1175.0 kJ/mol with TLR4. V2 interactions with the TLR2 included 10 residues, forming a network of 12 hydrogen bonds (**Figure 5A**). With TLR4, the interaction of V2 involved 9 residues, forming 10 hydrogen bonds (**Figure 5B**). Among these interacting residues 7 were glycine residues (**Supplementary Table 8**). This suggests the strong interactions interface that could be crucial for triggering an immune response. For V3, the interactions with TLR2 and TLR4 also offered insights with the lowest energies of -1133.5 kJ/mol and -1105.2 kJ/mol respectively. The interaction with TLR2 encompassed 8 residues, forming 10 hydrogen bonds (**Figure 6A**). On the TLR4 side, V3 interactions involved only 5 residues, forming a modest 5 hydrogen bonds (**Figure 6B**). Similar to the case of V2, glycine of V3 was mostly involved in the interactions with TLR4 (**Supplementary Table 8**). While fewer in number, these interactions could still have functional significance in immune response modulation. All three vaccine constructs were found to be engaged with TLR2 and TLR4 in unique ways. V1 demonstrated robust interactions with both receptors while V2 and V3 exhibited interactions with fewer hydrogen bonds.

Interestingly, Asn414, Lys480, Ile304, and Ser333 of TLR2 interacted with all three vaccine constructs. Phe325 and Tyr326 of TLR2 were present with both V1 and V2 while Lys360 of TLR2 was present with both V2 and V3. Glu42 and Lys186 of TLR4 occurred twice in interaction with V2 and V3. Moreover, Gly411, Gly421, Ala422, and Glu425 of V1; Lys299 and Gly323 of V2 and Ala442 of V3 were repeated in interactions with both receptors (**Supplementary Table 8**). These frequent interactions suggest a level of convergence between the vaccine constructs and TLR2/TLR4 receptors and might play a key role in the formation of hydrogen bonds and amplification of molecular interactions with the receptors.

Normal Mode Analysis of the vaccine V1 was performed using IMODs server to confirm its stability. The results indicate the deformability, B-factor, Eigenvalue, covariance matrix, and elastic network in the form of graphs. These graphs depict the stability of the vaccine-receptor complexes. The graphs representing major differences among the results of vaccine V1 with TLR2 and TLR4 are the deformability, covariance matrix, and elastic network. Deformability tells about a molecule’s ability to modify its residues. And it is indicated in the form of peaks. The higher the peak, the higher the deformability (**Supplementary Figures 4A, D**). The covariance matrix describes the relationships between the complicated residues. A complex is stronger if its residues are highly correlated. Blue, white, and red colors were used to indicate the residues that had anticorrelations, no correlation, and varied correlation, respectively, in the (**Supplementary Figures 4B, E**). Another parameter, the elastic network model, illustrates that the springs that are used to connect the pair of atoms together are represented by dots in graphs, which are colored on the basis of the stiffness of the springs. The stiffer the spring, the darker the color of the dots (**Supplementary Figures 4C, F**).

### Molecular dynamic simulations

**Figure 7:**
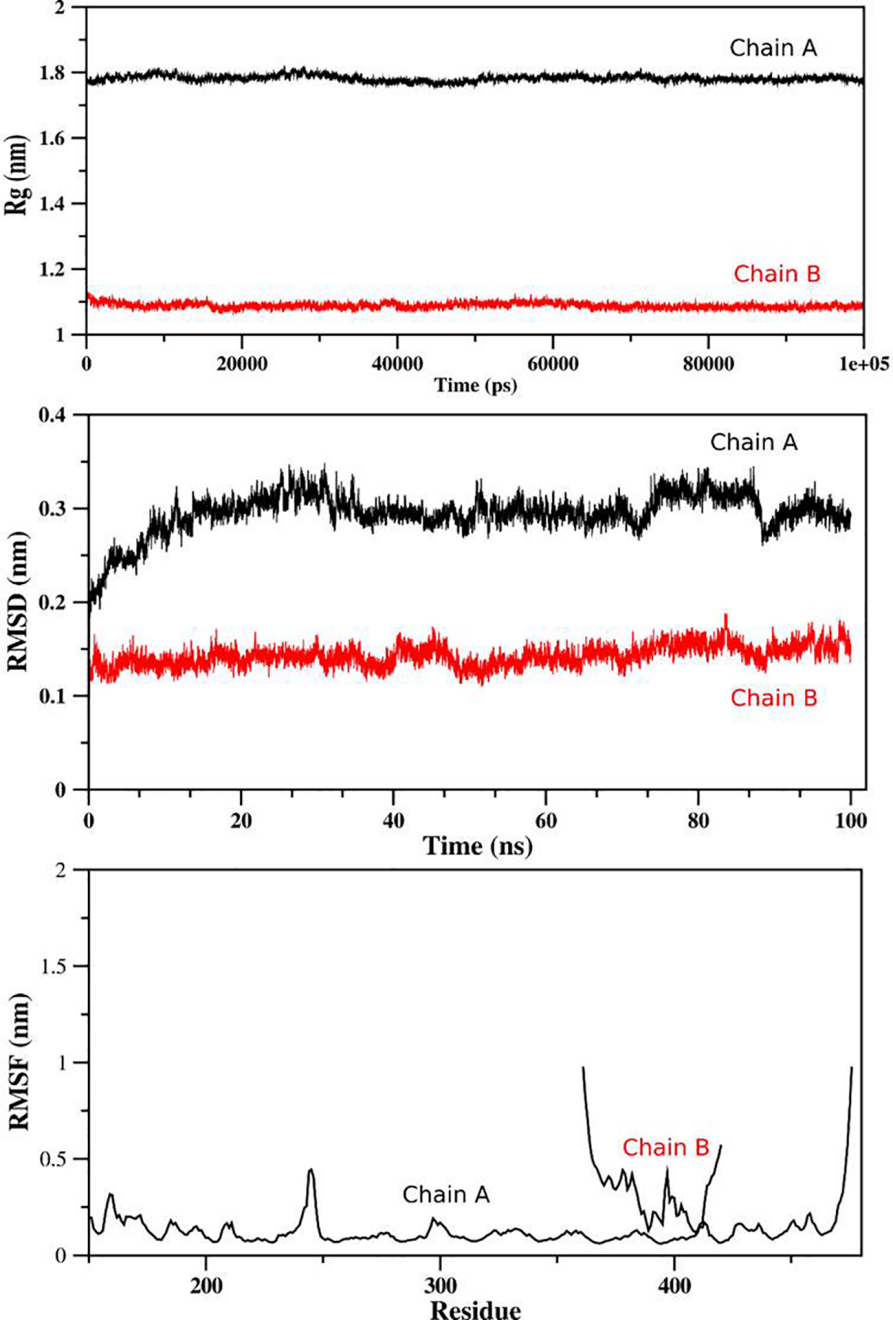
The analysis of MD simulation trajectory of V1-TLR2 complex: The MD post simulation analysis was carried out using GROMACS scripts. The receptor was Protein Chain A while Chain B was the incorporated ligand. The RMSD, RMSF and radius of gyration was plotted as function of residues number and time (ns) for both chains. The PDB (3FXI) was used as initial structure for receptor (chain A) while ligand (chain B) was docked using MOE suit for complex generation as per standard protocol of MOE and GROMACS.

**Figure 8:**
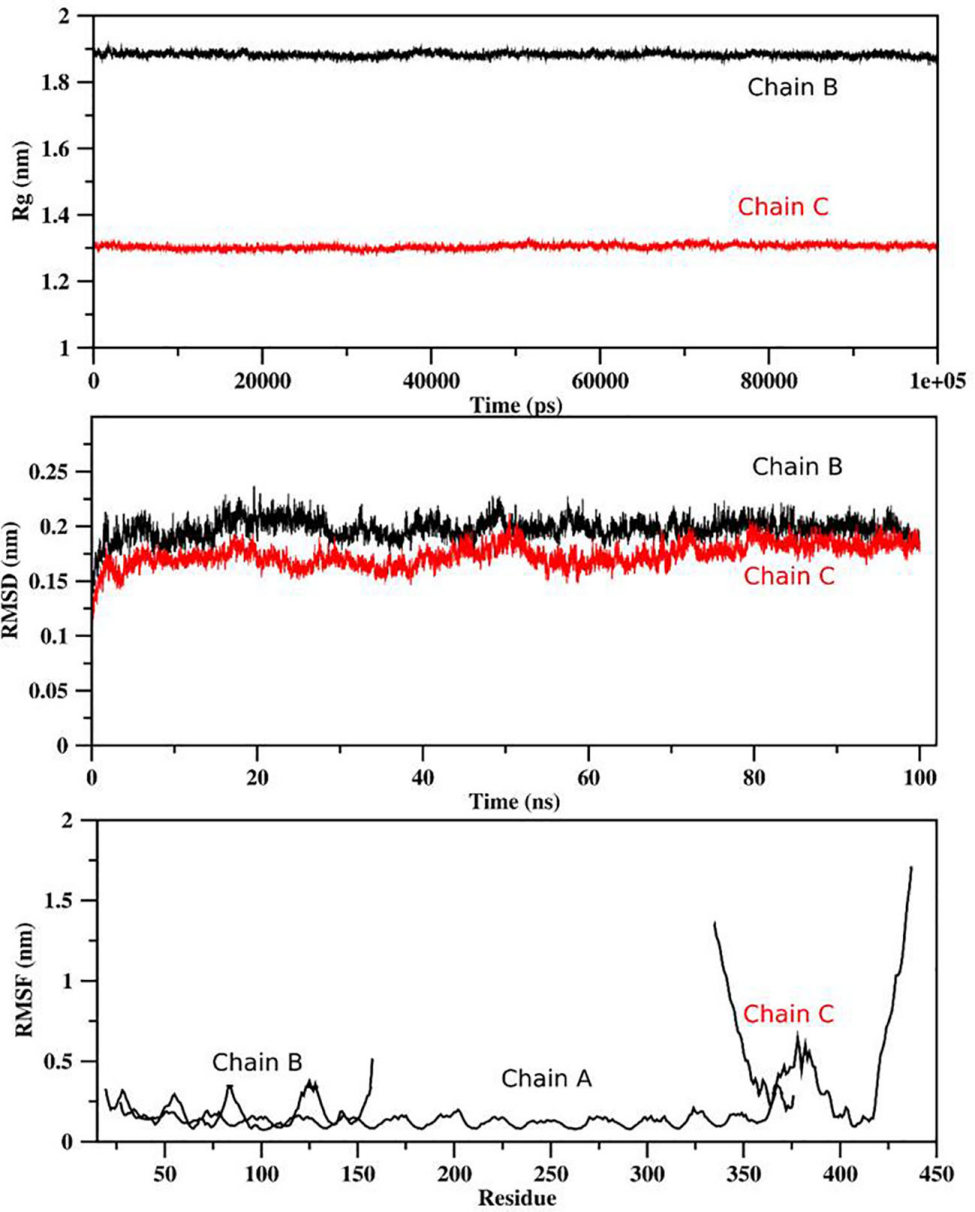
The analysis of MD simulation trajectory of V1-TLR4 complex: The MD post simulation analysis was carried out using GROMACS scripts. The receptors were protein chain A and Protein Chain B while Protein Chain C was ligand. The RMSD, RMSF and radius of gyration of all the proteins chains were plotted as function of residues number and time (ns). The PDB (2Z7X) was used as initial structure while ligand peptide was docked using MOE suit for complex generation as per standard protocol of MOE and GROMACS.

**Figure 9:**
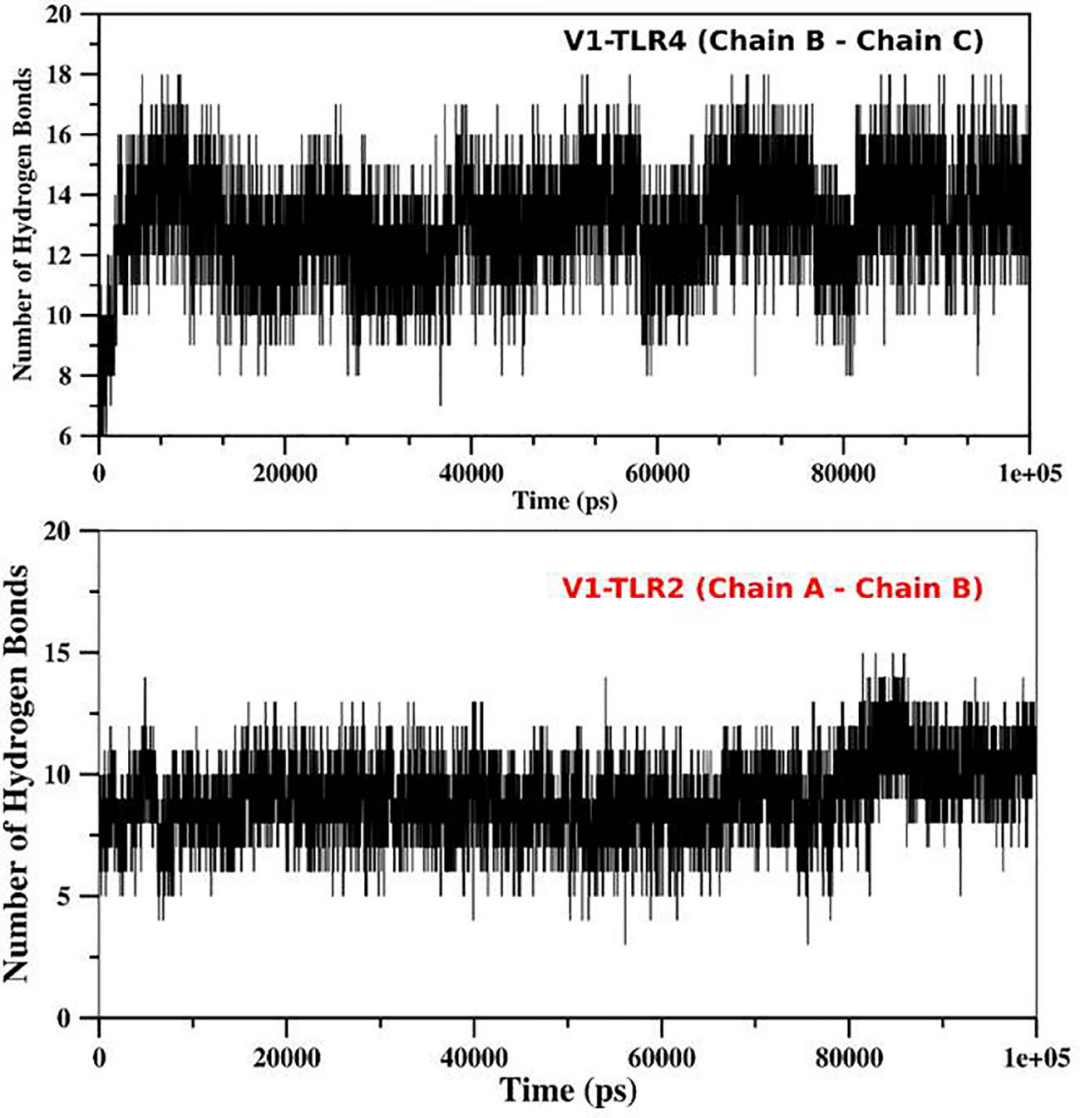
The Hydrogen bond analysis: The hydrogen bond analysis of V1-TLR2 and V1-TLR4 was obtained from md trajectory and plotted as function of time (ps). The interaction between different chains of the both complexes were analyzed and their labeled as mentioned in the figure.

**Figure 10:**
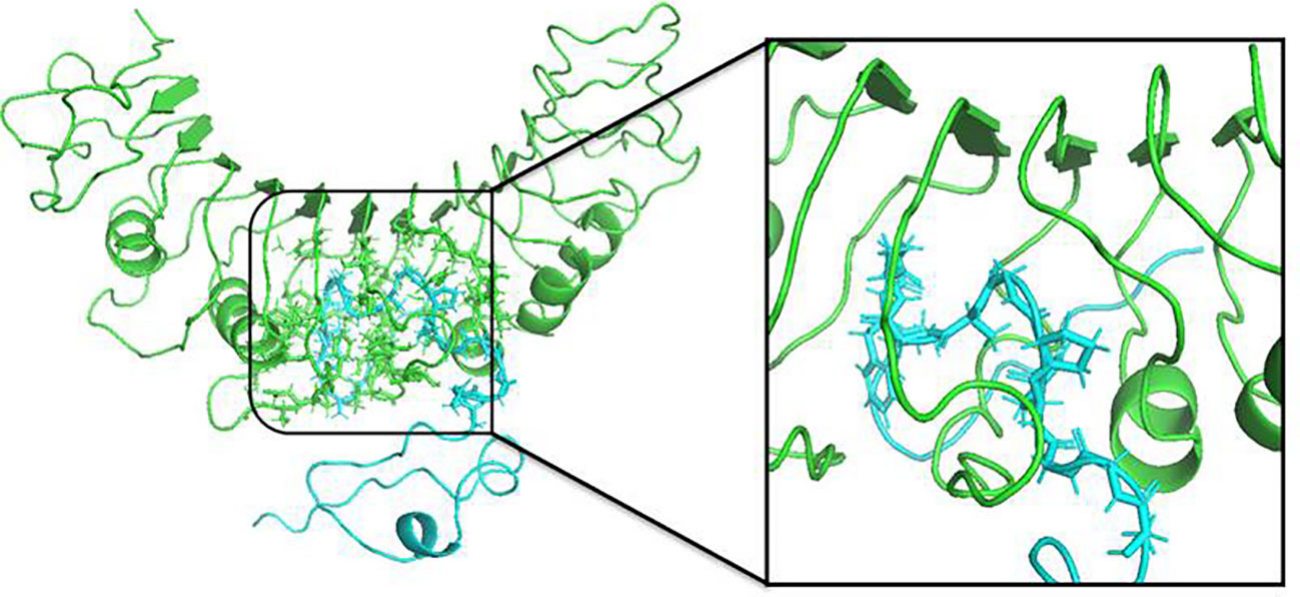
The structure of V1-TLR2 complex after MD simulation for 100 ns was obtained using GROMACS. The insertion of the protein chain B (ligand) is shown in color Cyan complexed with TLR2 (green) and interacting residues were shown in stick form.

**Figure 11:**
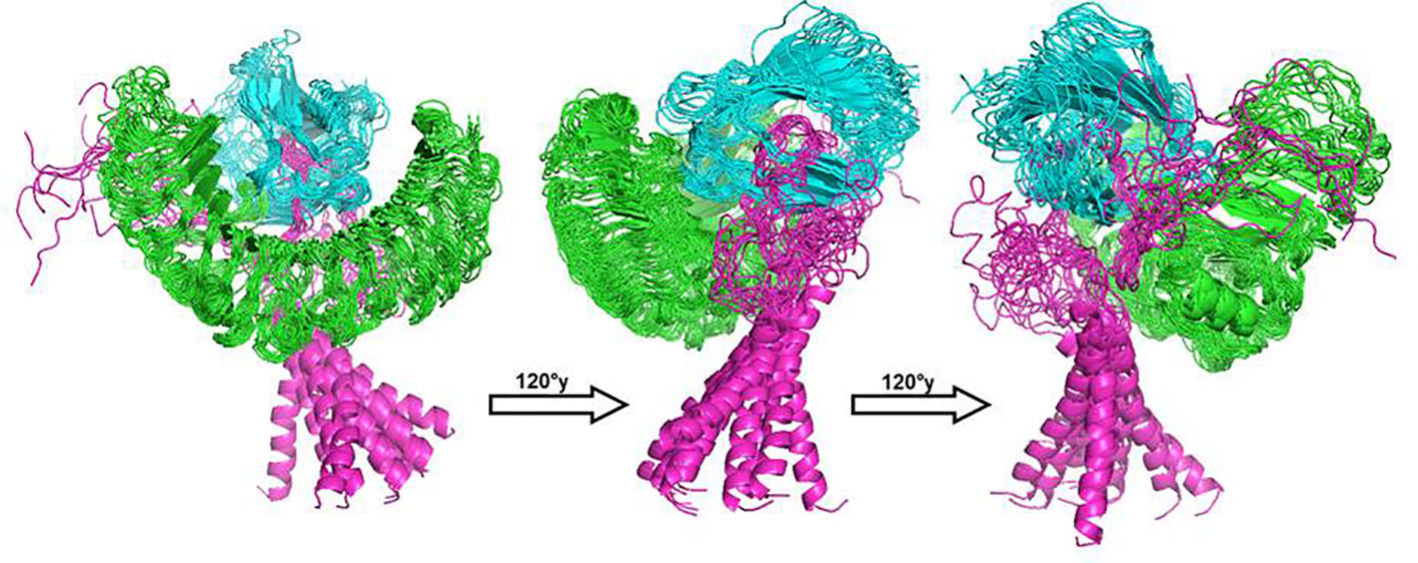
The structure of V1-TLR4 complex after MD simulation for 100 ns was obtained using GROMACS. The receptor chains (A and B) were shown with colors green and cyan respectively while ligand (V1) considered as chain C was labelled with color magenta. The structure of all protein was extracted after time interval of 5 ns and grouped in software Pymol for brief analysis.

For determination of the stability between the final potential vaccine construct i.e., V1 with the human immune receptors, the MD simulation was carried for 100 ns using standard protocol of GROMACS (24). The **Figure 7** shows the results obtained for V1-TLR2 where radius of gyration (Rg), RMSD and RMSF analysis of both proteins chains as function of time (ns) and residues, respectively, were analyzed. The overall behavior was found stable during the course of simulation trajectory. Similarly, the Radius of gyration (Rg), RMSD and RMSF of V1-TLR4 for interacting protein chains (B and C) was analyzed and shown in **Figure 8**. The complex formation between protein chain A and B was stabilized by hydrogen bonding in the range of ≤ 3 Å. The hydrogen bond analysis as function of time was analyzed and plotted (**Figure 9**). The MD trajectory was transformed into the ensemble analysis and structure after each 5 ns was extracted from the trajectory. The ensemble analysis of complexes is important for the recognition and target active site and ligands stability as function of time (**Figures 10**, **11**).

### Host immune response

The C-ImmSim server provides the immune profile of vaccine V1. During immune simulations, vaccines are injected in doses. Mostly, three injections are applied at different time intervals, and as a result, immunogenicity is increased, reaching its maximum after the third dose. Therefore, we injected our vaccine in three doses at the time steps of 01, 84, and 170. The results indicated that the chances of danger were too low (**Supplementary Figure 5A**). Our constructs produced several distinct antibodies, including IgG, IgG1, IgG2, and IgM. Furthermore, the percentage of IgM and IgG increased instantly after the third dose of injection (**Supplementary Figure 5B**). The number of activated T-cells and B-cells also increased after the vaccine injections (**Supplementary Figures 5C, D**). All these results indicated that the designed vaccine V1 is highly immunogenic.

### Codon optimization and *in-silico* cloning

Before *in silico* cloning, the codon optimization of the vaccine construct was performed using the Jcat server. The CAI and GC-content value of the final construct was found to be 0.98 and 48.89. The sequence of vaccine V1 before and after codon adaptation are shown in **Supplementary Figure 6**. The cloning was performed by inserting the vaccine into the pet-28(+) after the codon adaptation of the vaccine sequence. The clone formed after cloning is indicated in **Supplementary Figure 7**.

## Discussion

Vaccination is a successful method for preventing a number of fatal diseases and is widely used worldwide (52). Early vaccinations employed weak organisms, as in the case of cowpox or horsepox (53). Although these vaccinations led to minor illnesses while supporting the body’s development of a robust immune system, the host may be at a high risk for infection. Another vaccine employs germs that have already died, but it needs several shots to produce a powerful immunity. These vaccinations are dangerous and have a history of spreading disease (54). There is a demand for new vaccines with higher safety ratings. One of the recent and safer methods for vaccine development is its computational formulation using various tools of immunoinformatics. The “price-and-period” strategy of conventional vaccines is challenged by computational vaccinology, which hold the potential to transform vaccine development (55). There has been a surge in the use of computational methodologies for vaccine production based on genomic information, which has been dubbed “reverse vaccinology” due to the massive intake of genome sequence information from numerous pathogens and diverse hosts (56). Several studies have been reported using reverse vaccinology and immunoinformatics techniques for novel targets identifications and vaccine designing (57–64).

Peptide vaccines are a type of vaccine and are known to induce broad-spectrum immunity against the different forms or strains of a pathogenic organism (65). Immunological memory from vaccinations can last for several years to several decades. Any successful vaccination method is thought to require the stimulation of both B and T lymphocyte-mediated immune responses because this can result in a quicker and more effective immune response when the host comes into contact with the target pathogen at any time of his/her life (66). In order to produce an immune response that results in the creation of memory T and B cells, the body is made to believe by the vaccination that it has been attacked by a pathogen (67). The successful identification of specific target antigens, or more specifically, portions of the specific antigens termed epitopes, is necessary for the development of effector and memory T and B cells. Therefore, when it comes to the development of vaccines, it is very important to predict T and B cell epitopes on target antigens.

Six proteins namely, Nf314 (virulence-related protein), Hsp70 (involved in pathogenicity), nfa1 (necessary for locomotion and food cup formation), MP2CL5 (involved in pathogenicity), Nf23 (involved in virulence), and Actin-1 (involved in pathogenicity) were selected based on previous literature (68–73). The selected proteins were reported to possess the required potential to be used for the vaccine designing. The proteins used for the prediction of epitopes in this study were the heat shock protein (Hsp70) (Uniprot ID: Q6B3P1) and a 23-kda protein (Nf23) (Uniprot ID: A0A6A5CB91) of *N. fowleri*. Heat shock proteins (HSPs) produced by parasites are crucial for the adaptive responses necessary for parasite survival. Thermophily is a special trait of pathogenic *N. fowleri* (69). While nonpathogenic Naegleria spp. cannot sustain high temperatures, *N. fowleri* can tolerate high temperatures, indicating that HSPs in the amoeba are critical for survival against changes in temperature and may even be crucial for the virulence of the amoeba. HSPs of parasitic organisms take part in a number of vital procedures, including the regulation of the host’s immune system, protein folding, and the refolding of denatured proteins (69). Therefore, targeting the heat shock protein of *N. fowleri* is very helpful while formulating a vaccine against it because its inhibition can lead to the death of *N. fowleri*. Nf23 (23-kda protein) is a membrane protein and has potential interactions with other membrane proteins. Previous studies on Nf23 indicated that it plays many important functions, in the virulence of *N. fowleri*, as its structural characteristics revealed that it might operate as a lipid-binding protein, transmembrane receptor, and signaling receptor (72). This indicated that this protein is very beneficial for *N. fowleri* because its inhibition can paralyze this parasite, indicating its potential as a dominant vaccine target.

The immune response to viral infections must include CTL-mediated cytotoxic activity in order to be effective. Some of the viral proteins are broken down by cells infected with the virus and then presented to CTLs together with MHC class I molecules. Through the release of cytotoxic granules, CTLs that recognize the degraded portions of viral proteins known as epitopes kill infected cells (74). One CTL epitope has been isolated in this study after filtering through several factors in order to build a vaccine against *N. fowleri*. HTLs are activated when antigen-presenting cells present viral particles to them together with MHC class II molecules. The HTLs release a variety of chemokines and cytokines, including IFN-gamma, IL-4, and IL-10, which have different functions in the immunological response to invaders (75). Most effector T lymphocytes, that is, CTLs and HTLs, die once the antigens are eliminated, although a small percentage do so and form the memory T cell reservoir (76). Our investigation led to the selection of six HTL epitopes for vaccine development.

B lymphocytes bind to surface-exposed antigenic epitopes of target cells using B cell receptors, which are essentially membrane-bound immunoglobulins, and then incorporate, process, and present them to T cells (77). The processed epitopes are exposed on the surface of B cells together with MHC class II and are recognized by HTLs that have a corresponding T-cell receptor (TCR). As a result, B lymphocytes become plasma cells that secrete antibodies (78). The ability of these antibodies to neutralize infections is crucial (79). A different possibility is that the activated B lymphocytes start germline center processes, which produce memory B cells and long-lived plasma cells (80). Eight B-cell epitopes have been found to be suitable for the vaccine design in this study.

After the epitope prediction, the vaccine was formed by joining the epitopes via linkers. Peptide vaccines are generally small in size and thus not immunogenic by themselves. Therefore, to increase their immunogenicity, it is necessary to add adjuvants (65). Three different adjuvants, namely, L7/L12 Ribosomal-protein adjuvant, Beta-defensin adjuvant, and Granulocyte-macrophage colony adjuvant, were used in this study to form three vaccine constructs with varying immune profiles. All the constructs formed were antigenic, non-allergenic, and highly immunogenic. The secondary and tertiary structure prediction in this study was done by Psipred and SWISS-model severs. The secondary structures provide information about the alpha and beta helix, and random coils of the designed vaccines. The predicted tertiary structure was refined and validated by the Ramachandran plot. This plot provides information about the number of amino acid residues of vaccines in the most favored regions, allowed regions, generously allowed regions, and disallowed regions, which is useful for determining the efficacy of the construct’s tertiary structures. All our constructs showed a sufficient number of residues in the most favored regions (>90%), which validated their efficacy.

A vital bioinformatics tool that is frequently used to forecast the binding affinity and posture between a ligand and its matching receptor is Molecular Docking (81). Molecular docking has proven crucial not only to computational drug design but also to studies on vaccine design. Molecular docking is an essential tool in immunoinformatics for simulating the interaction of T lymphocyte epitopes with their respective MHC molecules (82). The binding energy predicts the affinity of the ligand to bind to the receptor. The lower the binding energy, the stronger the protein-ligand complex. The vaccine constructs V1, V2, and V3 were docked with the Human immune receptors. The Toll-like Receptors (TLRs) are the important immune receptors in humans and are essential for the innate immunity. They identify both pathogen and damage-associated molecular patterns, and have been implicated in a number of autoimmune and inflammatory illnesses (83). The toll-like receptors used in this study were the TLR2 and TLR4 because many studies have used these receptors for vaccine-receptor docking purposes (23, 24, 34, 84). The vaccine V1 showed the best results and was preceded further. Normal mode analysis and molecular dynamic simulations provide information about the stability of the vaccines. The results of the both indicated the stability of the V1-receptors complexes. The increase in the immune responses of the constructs after several doses of vaccines was determined by immune simulations. Humoral immunity is produced when B- and T-cell activity is higher and B-cell memory persists for several months, which is crucial for enhancing the immune response. The IgM+IgG antibodies were also increased after the two doses of injection, and the ability of our vaccine was shown to have a very low risk of danger. Overall, immune simulations represented the potential of our vaccine to cause immunogenicity in individuals. Furthermore, *in silico* cloning represented the potential of our vaccine to have a good expression probability in host organisms.

In the case of an outbreak of *N. fowleri*, the production of vaccines containing weak or dead pathogens is not a good choice because it takes much time and their efficacy is also not that great to fully protect individuals against the virulence of *N. fowleri*. Therefore, the computational formulation of a peptide vaccine against this pathogen will be a very quick, free, and efficient method. Therefore, our immunoinformatics based multi-epitope subunit peptide vaccine can be beneficial to protect mankind against *N. fowleri.* Additional testing of its effectiveness *in vivo* and *in vitro* is recommended to prepare a potential vaccine against this brain eating parasite.

## Conclusion

The formation of multi-epitope peptide vaccines against such parasites has not gained much success till now. These vaccines are gradually displacing conventional vaccines due to their well-being and logical design. They lack the entire pathogen and are only made up of the small antigenic parts of microorganisms. Owing to the fact that these vaccines can confer long-term protection and have a very low risk of side effects, multi-epitope vaccines can be administered to people with weakened immune systems. Potential immunodominant MHC-I, MHC-II, and B-cell epitopes were selected using reverse vaccinology methods in order to induce cellular and humoral immunity. The vaccine designed in this study might be suggested as an acceptable candidate against *N. fowleri* based on the immunological, structural, and physicochemical studies as well as evaluations. Additionally, docking and MD simulations were carried out, which demonstrated the vaccine’s stable binding to TLR2 and TLR4. In addition to having acceptable physicochemical properties, the suggested vaccine is anticipated to induce potent immune responses against *N. fowleri* due to the application of multiple epitopes and adjuvants. *In silico* cloning ensured that the designed vaccine could potentially be expressed in the microbial expression system, enabling quick scale-up of the vaccine in the event of a potential outbreak to combat *N. fowleri* infection. The effectiveness of this unique multi-epitope vaccine needs to be confirmed through *in vitro* and *in vivo* experimentation.

## Data availability statement

The original contributions presented in the study are included in the article/**Supplementary Material**. Further inquiries can be directed to the corresponding authors.

## Author contributions

MS: Conceptualization, Project administration, Resources, Supervision, Writing – review & editing. AS: Formal Analysis, Investigation, Methodology, Writing – original draft. TW: Data curation, Formal Analysis, Investigation, Writing – original draft. KC: Data curation, Visualization, Writing – review & editing. SA: Data curation, Formal Analysis, Investigation, Visualization, Writing – original draft. AZ: Formal Analysis, Investigation, Visualization, Writing – original draft. UN: Conceptualization, Methodology, Validation, Visualization, Writing – review & editing. AI: Formal Analysis, Investigation, Software, Writing – original draft. RU: Methodology, Software, Validation, Visualization, Writing – review & editing. EA: Data curation, Funding acquisition, Methodology, Validation, Writing – review & editing. SCO: Conceptualization, Data curation, Funding acquisition, Validation, Visualization, Writing – review & editing. S: Formal Analysis, Investigation, Methodology, Visualization, Writing – original draft.

## Supplementary material

Supplementary Figure 1 Secondary structure prediction of V1 **(A)**, V2 **(B)**, and V3 **(C)** indicating helixes, strands, and coils of the designed vaccines.

Supplementary Figure 2 Secondary structure of V1 **(A)**, V2 **(B)**, and V3 **(C)** indicating the polar, non-polar, aromatic, and hydrophobic residues of these vaccines.

Supplementary Figure 3 Residues involved in the formation of Conformational B-cell epitopes **(A)** Residues of V1 scoring 0.881 **(B)** Residues of V1 scoring 0.735 **(C)** Residues of V2 scoring 0.886 **(D)** Residues of V3 scoring 0.88 **(E)** Residues of V3 scoring 0.724.

Supplementary Figure 4 Normal mode analysis results of V1-TLR2 **(A)**-Deformability, **(B)**-Covariance matrix, C-Elastic network model) and V1-TLR4 (D-Deformability, E-Covariance matrix, F-Elastic network model).

Supplementary Figure 5 Immune simulation of vaccine V1 by C-ImmSim server. **(A)** Interleukins and cytokines profile. **(B)** Primary Antibody responses. **(C)** Active Cytotoxic T-cells profile. **(D)** Active B-cells profile.

Supplementary Figure 6 Codon usage of the vaccine V1 before and after adaptation.

Supplementary Figure 7
*In silico* cloning of the vaccine V1 performed by inserting the vaccine into the pet-28a(+) vector.
