# Corrigendum: Structural informatics approach for designing an epitope-based vaccine against the brain-eating *Naegleria fowleri*

**DOI:** 10.3389/fimmu.2023.1345435

**Published:** 2023-12-07

**Authors:** Asifa Sarfraz, Tehreem Ul Wara, Sheheryar, Ke Chen, Shahid Habib Ansari, Aqal Zaman, Umar Nishan, Anwar Iqbal, Riaz Ullah, Essam A. Ali, Mohibullah Shah, Suvash Chandra Ojha

**Affiliations:** ^1^ Department of Biochemistry, Bahauddin Zakariya University, Multan, Pakistan; ^2^ Department of Biochemistry and Molecular Biology, Federal University of Ceara, Fortaleza, Brazil; ^3^ Department of Infectious Diseases, The Affiliated Hospital of Southwest Medical University, Luzhou, China; ^4^ Department of Microbiology & Molecular Genetics, Bahauddin Zakariya University, Multan, Pakistan; ^5^ Department of Chemistry, Kohat University of Science & Technology, Kohat, Pakistan; ^6^ Department of Chemical Sciences, University of Lakki Marwat, Khyber Pakhtunkhwa, Pakistan; ^7^ Department of Pharmacognosy, College of Pharmacy, King Saud University, Riyadh, Saudi Arabia; ^8^ Department of Pharmaceutical Chemistry, College of Pharmacy, King Saud University, Riyadh, Saudi Arabia

**Keywords:** primary amoebic meningoencephalitis, parasite, epitope, brain-eating, *Naegleria*, immunoinformatics, reverse vaccinology

In the published article, there was an error regarding the affiliation for Suvash Chandra Ojha. Instead of “Department of Biochemistry and Molecular Biology, Federal University of Ceara, Fortaleza, Brazil”, it should be “Department of Infectious Diseases, The Affiliated Hospital of Southwest Medical University, Luzhou, China.

The authors apologize for this error and state that this does not change the scientific conclusions of the article in any way. The original article has been updated.

