# Supplementary figures and images for: Structural informatics approach for designing an epitope-based vaccine against the brain-eating Naegleria fowleri
Source: Front Immunol. 2023 Oct 30;14:1284621. doi: 10.3389/fimmu.2023.1284621 (PMC10642955; doi:10.3389/fimmu.2023.1284621)

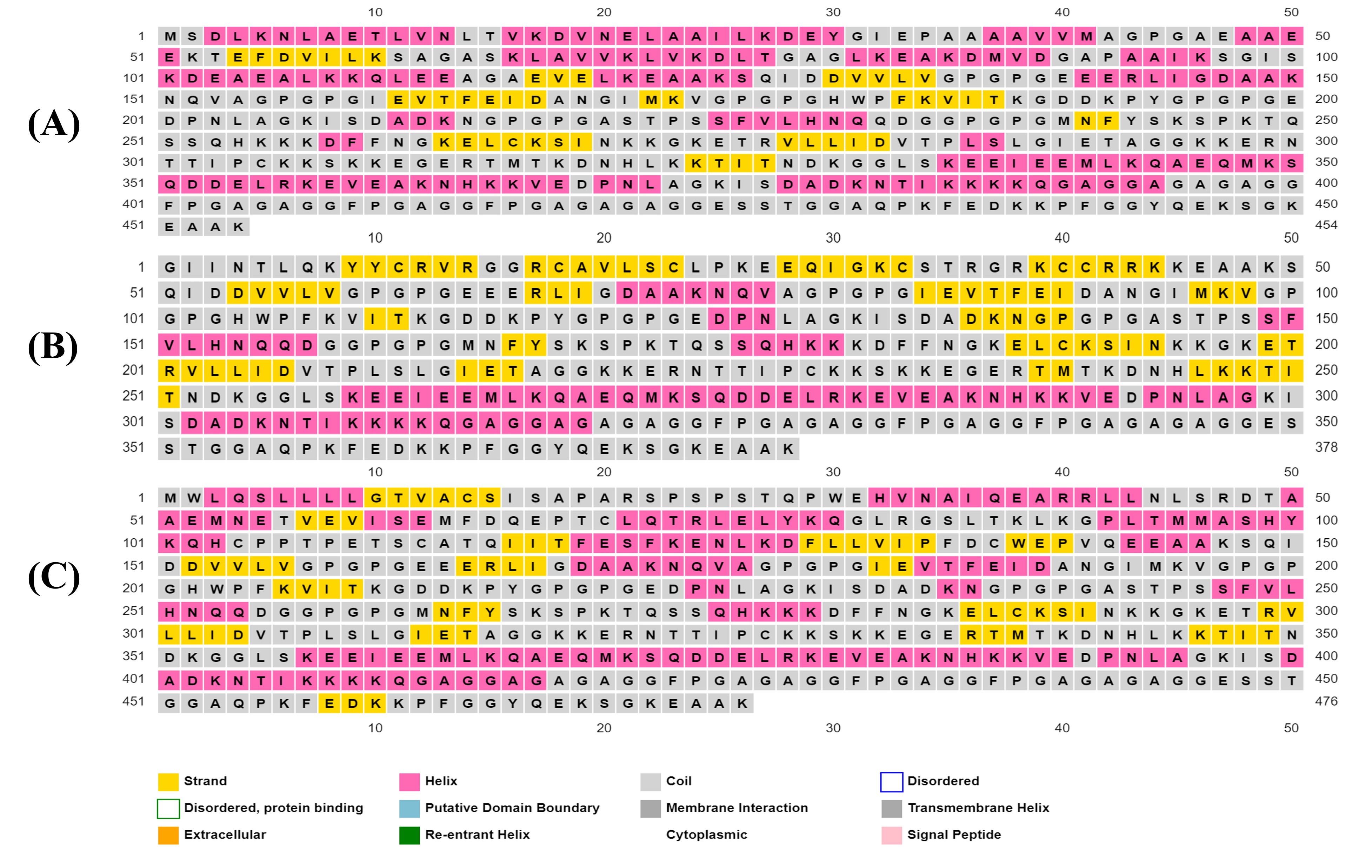

Supplement: Supplementary Figure 1 — Secondary structure prediction of V1 (A), V2 (B), and V3 (C) indicating helixes, strands, and coils of the designed vaccines. [file Image_1.jpeg]

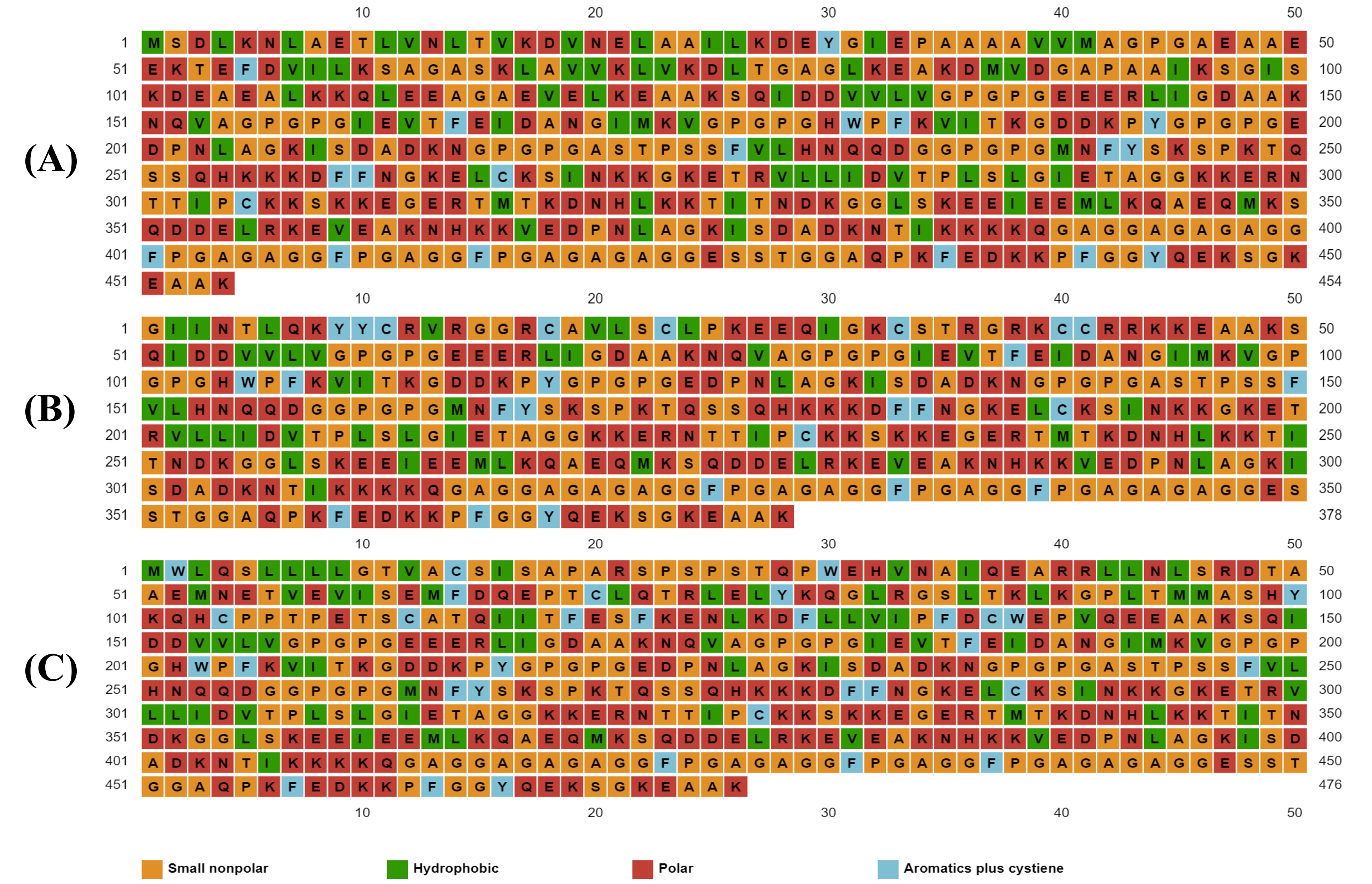

Supplement: Supplementary Figure 2 — Secondary structure of V1 (A), V2 (B), and V3 (C) indicating the polar, non-polar, aromatic, and hydrophobic residues of these vaccines. [file Image_2.jpeg]

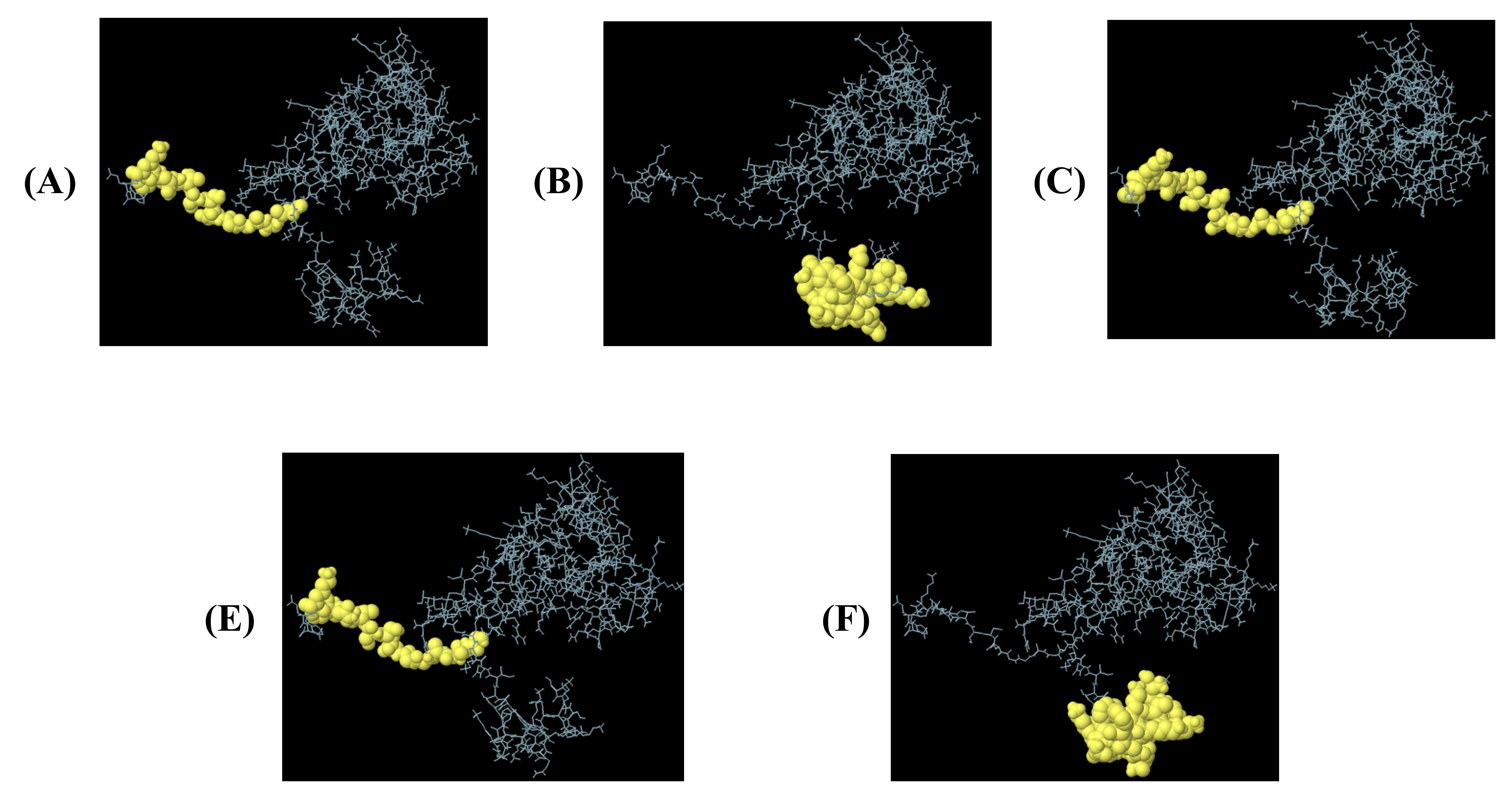

Supplement: Supplementary Figure 3 — Residues involved in the formation of Conformational B-cell epitopes (A) Residues of V1 scoring 0.881 (B) Residues of V1 scoring 0.735 (C) Residues of V2 scoring 0.886 (D) Residues of V3 scoring 0.88 (E) Residues of V3 scoring 0.724. [file Image_3.jpeg]

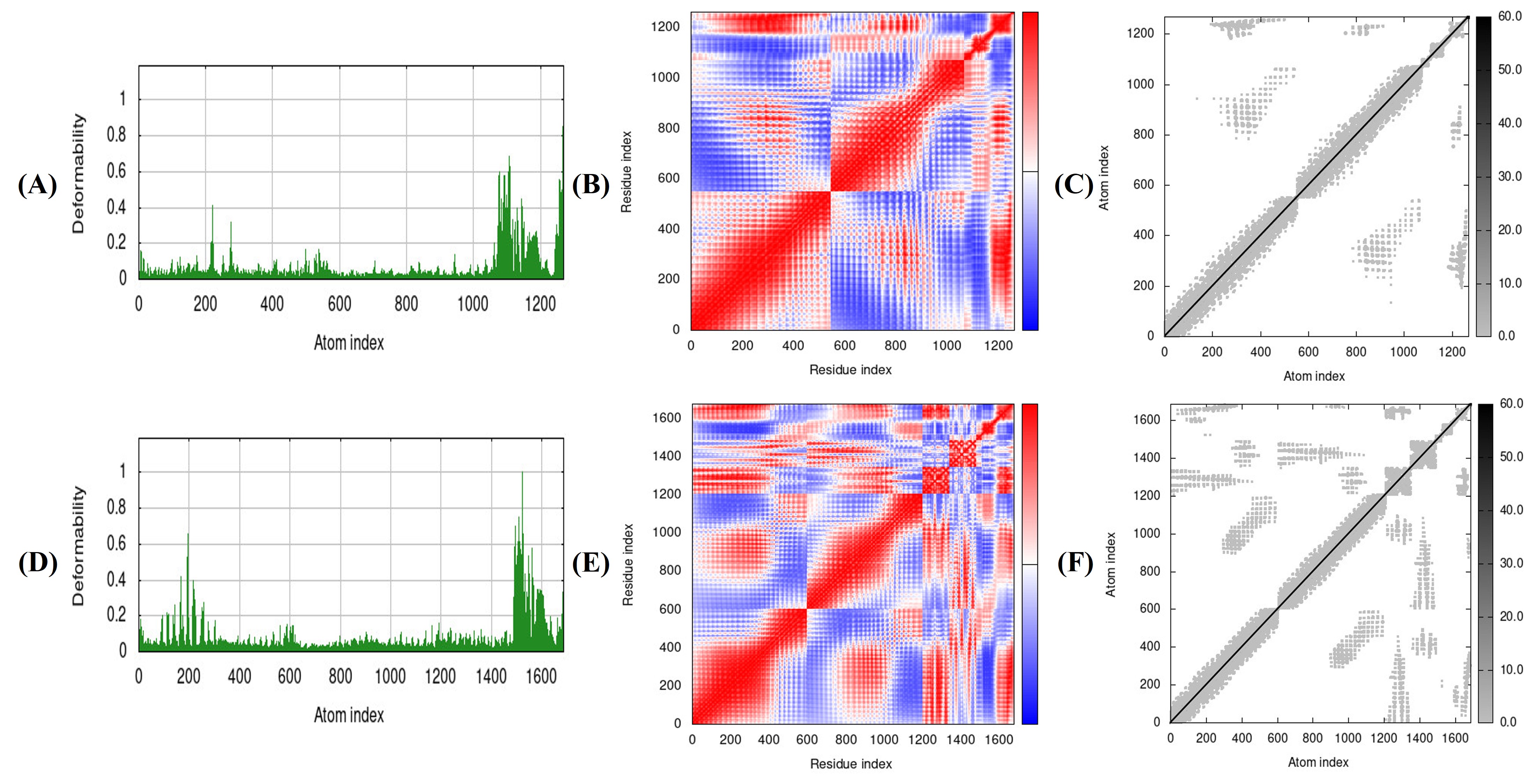

Supplement: Supplementary Figure 4 — Normal mode analysis results of V1-TLR2 (A)-Deformability, (B)-Covariance matrix, C-Elastic network model) and V1-TLR4 (D-Deformability, E-Covariance matrix, F-Elastic network model). [file Image_4.jpeg]

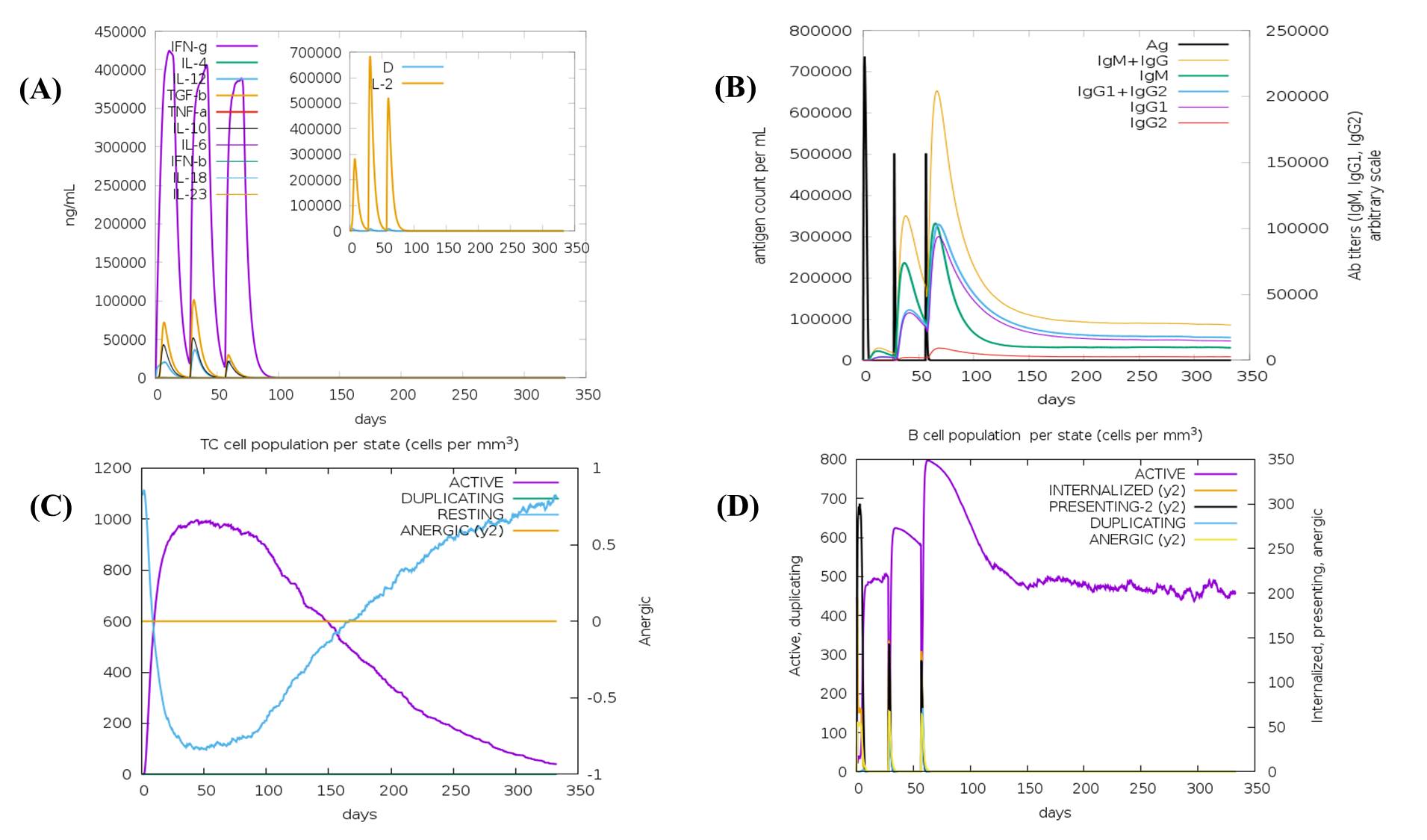

Supplement: Supplementary Figure 5 — Immune simulation of vaccine V1 by C-ImmSim server. (A) Interleukins and cytokines profile. (B) Primary Antibody responses. (C) Active Cytotoxic T-cells profile. (D) Active B-cells profile. [file Image_5.jpeg]

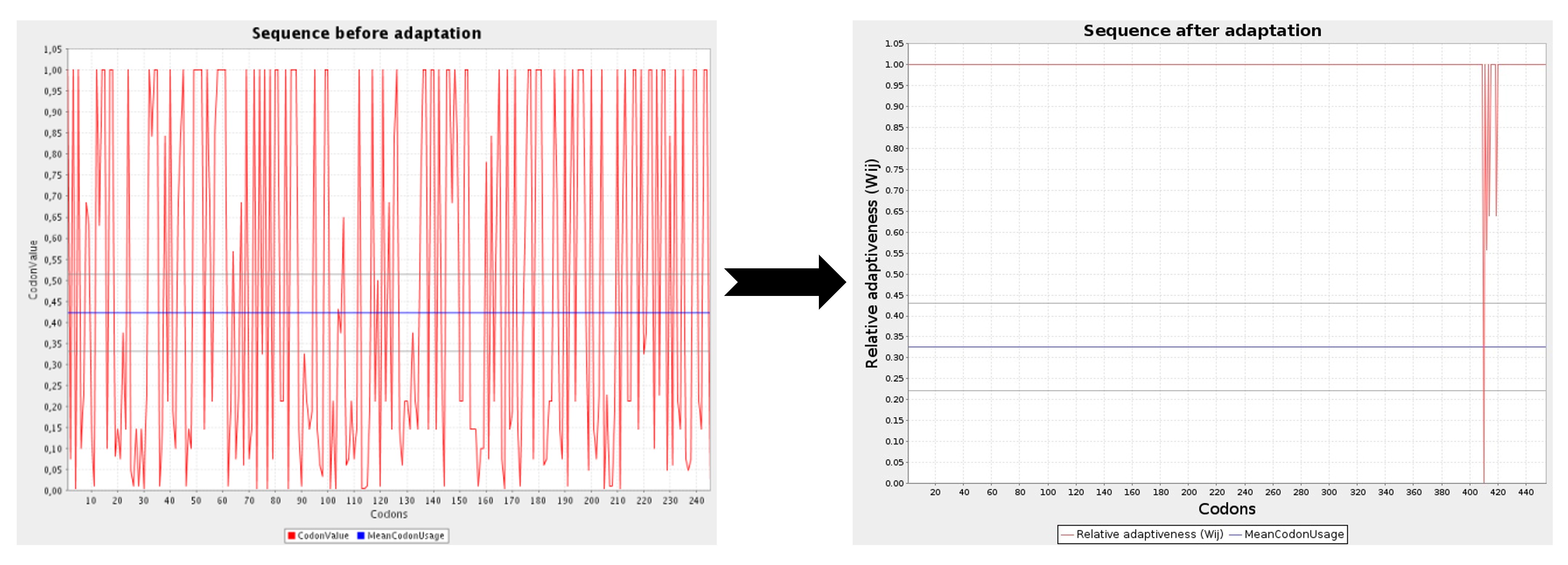

Supplement: Supplementary Figure 6 — Codon usage of the vaccine V1 before and after adaptation. [file Image_6.jpeg]

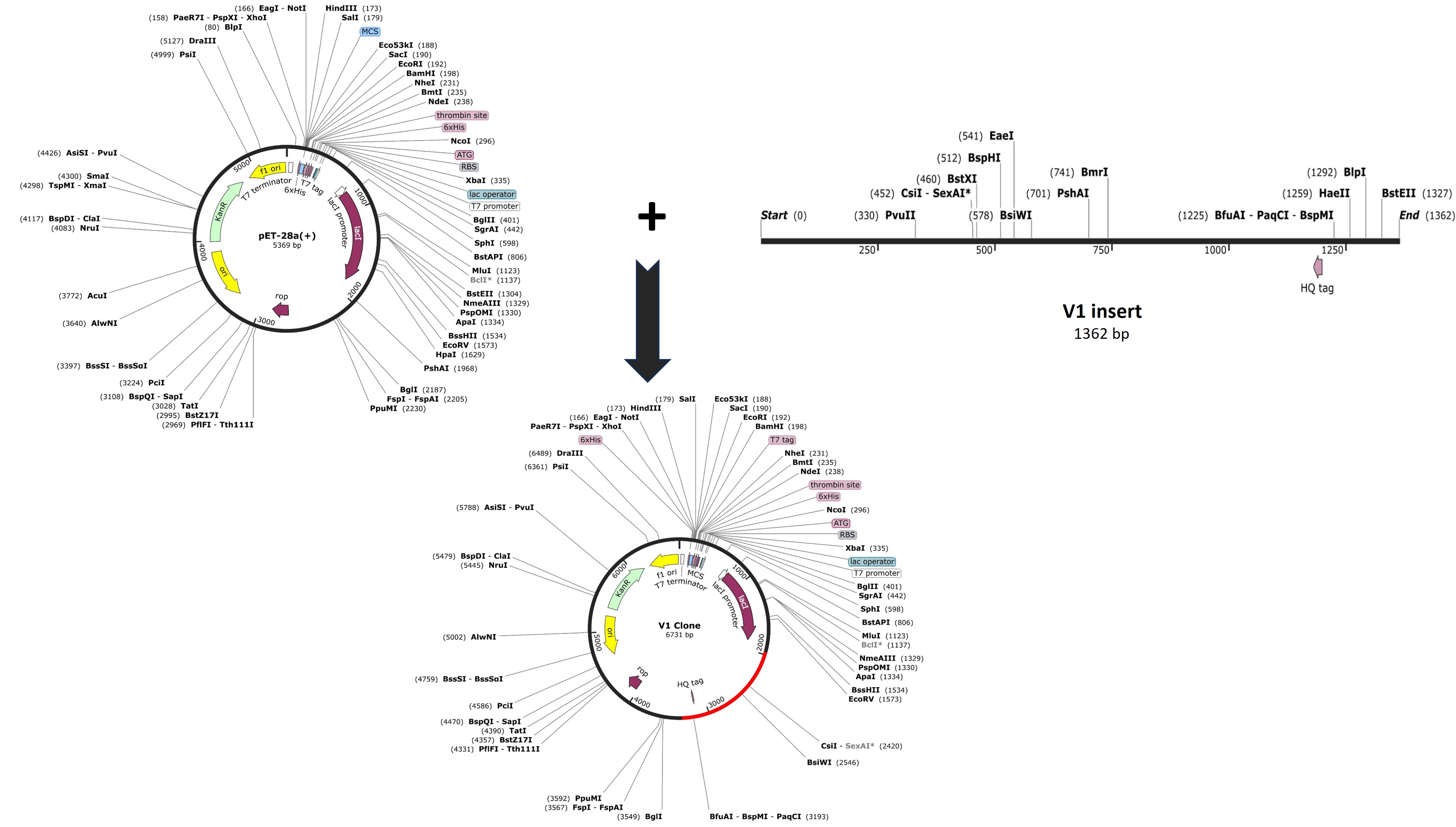

Supplement: Supplementary Figure 7 — In silico cloning of the vaccine V1 performed by inserting the vaccine into the pet-28a(+) vector. [file Image_7.jpeg]
