# Supplementary material for: Structural informatics approach for designing an epitope-based vaccine against the brain-eating Naegleria fowleri
Source: Front Immunol. 2023 Oct 30;14:1284621. doi: 10.3389/fimmu.2023.1284621 (PMC10642955; doi:10.3389/fimmu.2023.1284621)
Supplement: Supplementary file 8 [file Table_1.docx]

**Supplementary Table 1.**  Analysis & Selection of MHC-I target epitopes of Hsp70 (The row in bold shows the selected epitopes).

| **Sr No.** | **Allele** | **Start** | **End** | **Peptide** | **Score** | **Rank** | **Antigenicity** | **Allergenicity** | **Toxicity** | **Immunogenicity** |
| --- | --- | --- | --- | --- | --- | --- | --- | --- | --- | --- |
|  | **HLA-A*02:06** | **328** | **336** | **SQIDDVVLV** | **0.992551** | **0.01** | **Antigen** | **Non-allergen** | **Non-toxin** | **0.15434** |
|  | HLA-A*68:01 | 399 | 407 | ETAGGVMTK | 0.987282 | 0.01 | Antigen | Allergen | Non-toxin | -0.00686 |
|  | HLA-B*40:01 | 529 | 537 | KEVEAKNHL | 0.985006 | 0.01 | Antigen | Allergen | Non-toxin | -0.03731 |
|  | HLA-B*15:01 | 127 | 135 | KMRDIAEQY | 0.984088 | 0.01 | Non-antigen | Allergen | Non-toxin | 0.22237 |
|  | HLA-B*15:01 | 60 | 68 | AMNPHNTVF | 0.982755 | 0.01 | Antigen | Allergen | Non-toxin | 0.06903 |
|  | HLA-B*35:01 | 107 | 115 | EYKGETHTF | 0.940179 | 0.01 | Antigen | Allergen | Non-toxin | 0.14812 |
|  | HLA-A*02:03 | 36 | 44 | RTTPSYVAF | 0.880218 | 0.01 | Non-antigen | Allergen | Non-toxin | -0.10544 |
|  | HLA-A*02:01 | 176 | 184 | EPTAAAIAY | 0.965144 | 0.02 | Antigen | Allergen | Non-toxin | 0.26208 |
|  | HLA-B*07:02 | 540 | 548 | YAYQMKSTV | 0.910964 | 0.02 | Antigen | Non-allergen | Non-toxin | -0.6087 |
|  | HLA-A*11:01 | 373 | 382 | AVQAGVLTGK | 0.890342 | 0.02 | Antigen | Allergen | Non-toxin | 0.11709 |
