# Supplementary material for: Structural informatics approach for designing an epitope-based vaccine against the brain-eating Naegleria fowleri
Source: Front Immunol. 2023 Oct 30;14:1284621. doi: 10.3389/fimmu.2023.1284621 (PMC10642955; doi:10.3389/fimmu.2023.1284621)
Supplement: Supplementary file 9 [file Table_2.docx]

**Supplementary Table 2.** Analysis and selection of MHC-I target epitopes of Nf23.

| **Sr No.** | **Allele** | **Start** | **End** | **Peptide** | **Score** | **Rank** | **Antigenicity** | **Allergenicity** | **Toxicity** | **Immunogenicity** |
| --- | --- | --- | --- | --- | --- | --- | --- | --- | --- | --- |
|  | HLA-B*58:01 | 109 | 117 | LTFDNTVHW | 0.996798 | 0.01 | Non-antigen | Allergen | Non-toxin | 0.1443 |
|  | HLA-A*01:01 | 183 | 192 | FSRDPTNGMY | 0.984007 | 0.01 | Non-antigen | Allergen | Non-toxin | -0.01397 |
|  | HLA-A*03:01 | 18 | 26 | RIGPFLRYK | 0.973034 | 0.01 | Antigen | Allergen | Non-toxin | 0.14492 |
|  | HLA-B*08:01 | 133 | 141 | DVFTRVHPL | 0.920212 | 0.01 | Non-antigen | Allergen | Non-toxin | 0.18714 |
|  | HLA-A*32:01 | 75 | 83 | TQNGHHFTW | 0.850972 | 0.01 | Non-antigen | Allergen | Non-toxin | 0.21543 |
|  | HLA-B*07:02 | 120 | 128 | KPSSDHNQL | 0.955258 | 0.03 | Antigen | Allergen | Non-toxin | -0.24126 |
|  | HLA-A*03:01 | 90 | 99 | RVATGIAVPK | 0.90389 | 0.03 | Non-antigen | Allergen | Non-toxin | 0.27955 |
|  | HLA-B*35:01 | 217 | 225 | IPLSNGDVF | 0.907526 | 0.04 | Non-antigen | Allergen | Non-toxin | -0.10163 |
|  | HLA-B*08:01 | 206 | 214 | LLKAVTNSL | 0.773264 | 0.04 | Non-antigen | Allergen | Non-toxin | -0.05601 |
|  | HLA-A*68:02 | 47 | 55 | QTMITVPQV | 0.776693 | 0.05 | Non-antigen | Allergen | Non-toxin | 0.07654 |
