# Supplementary material for: Structural informatics approach for designing an epitope-based vaccine against the brain-eating Naegleria fowleri
Source: Front Immunol. 2023 Oct 30;14:1284621. doi: 10.3389/fimmu.2023.1284621 (PMC10642955; doi:10.3389/fimmu.2023.1284621)
Supplement: Supplementary file 10 [file Table_3.docx]

**Supplementary Table 3.** Analysis and selection of MHC-II target epitopes of Hsp70 (The row in bold shows the selected epitopes).

| **Allele** | **Start** | **End** | **Peptide** | **Rank** | **Antigenicity** | **Allergenicity** | **IFN-inducers** | **IL4 pred** | **IL10 pred** |
| --- | --- | --- | --- | --- | --- | --- | --- | --- | --- |
| HLA-DQA1*01:02/DQB1*06:02 | 365 | 379 | DEAVAYGAAVQAGVL | 0.01 | Antigen | Non-allergen | Negative | Non-inducer | Non-inducer |
| HLA-DRB1*08:02 | 310 | 324 | RNCFEPVKKVLQDSG | 0.01 | Non-antigen | Non-allergen | Negative | Inducer | Non-inducer |
| HLA-DRB1*04:01 | 570 | 584 | EEAIAYIDNNPSASK | 0.01 | Non-antigen | Non-allergen | Positive | Inducer | Non-inducer |
| HLA-DRB1*15:01 | 432 | 446 | AVTIQVYEGERTMTK | 0.03 | Non-antigen | Non-allergen | Negative | Inducer | Non-inducer |
| **HLA-DPA1*02:01/DPB1*14:01** | **46** | **60** | **EEERLIGDAAKNQVA** | **0.04** | **Antigen** | **Non-allergen** | **Positive** | **Non-inducer** | **Non-inducer** |
| HLA-DRB4*01:01 | 23 | 37 | GDNVEIIPNDQGNRT | 0.07 | Non-antigen | Allergen | Negative | Non-inducer | Non-inducer |
| **HLA-DRB3*01:01** | **469** | **483** | **IEVTFEIDANGIMKV** | **0.07** | **Antigen** | **Non-allergen** | **Negative** | **Inducer** | **Non-inducer** |
| **HLA-DRB1*08:02** | **89** | **103** | **HWPFKVITKGDDKPY** | **0.09** | **Antigen** | **Non-allergen** | **Negative** | **Inducer** | **Non-inducer** |
| **HLA-DQA1*01:02/DQB1*06:02** | **549** | **563** | **EDPNLAGKISDADKN** | **0.1** | **Antigen** | **Non-allergen** | **Negative** | **Inducer** | **Non-inducer** |
| HLA-DRB3*02:02 | 406 | 420 | TKLIERNTTIPCKKS | 0.1 | Non-antigen | Non-allergen | Positive | Inducer | Non-inducer |
