# Supplementary material for: Structural informatics approach for designing an epitope-based vaccine against the brain-eating Naegleria fowleri
Source: Front Immunol. 2023 Oct 30;14:1284621. doi: 10.3389/fimmu.2023.1284621 (PMC10642955; doi:10.3389/fimmu.2023.1284621)
Supplement: Supplementary file 11 [file Table_4.docx]

**Supplementary Table 4.** Analysis and selection of MHC-II target epitopes of Nf23 (The rows in bold show the selected epitopes).

| **Allele** | **Start** | **End** | **Peptide** | **Rank** | **Antigenicity** | **Allergenicity** | **IFN-inducers** | **IL4-inducers** | **IL10 inducers** |
| --- | --- | --- | --- | --- | --- | --- | --- | --- | --- |
| HLA-DPA1*01:03/DPB1*02:01 | 190 | 204 | GMYIVFINFHNNGQD | 0.1 | Antigen | Non-allergen | Negative | Non-inducer | Non-inducer |
| HLA-DRB1*04:05 | 21 | 35 | PFLRYKATDTDHDRS | 0.11 | Non-antigen | Non-allergen | Positive | Inducer | Non-inducer |
| **HLA-DQA1*04:01/DQB1*04:02** | **162** | **176** | **ASTPSSFVLHNQQDG** | **0.15** | **Antigen** | **Non-allergen** | **Positive** | **Non-inducer** | **Non-inducer** |
| HLA-DPA1*02:01/DPB1*01:01 | 96 | 110 | AVPKVNSSEFAVVLT | 0.19 | Non-antigen | Non-allergen | Negative | Inducer | Non-inducer |
| HLA-DPA1*01:03/DPB1*02:01 | 188 | 202 | TNGMYIVFINFHNNG | 0.49 | Antigen | Non-allergen | Negative | Non-inducer | Non-inducer |
| HLA-DQA1*01:02/DQB1*06:02 | 209 | 223 | AVTNSLASIPLSNGD | 0.54 | Non-antigen | Non-allergen | Negative | Non-inducer | Non-inducer |
| HLA-DQA1*04:01/DQB1*04:02 | 132 | 146 | SDVFTRVHPLVAEAP | 0.58 | Non-antigen | Non-allergen | Positive | Non-inducer | Inducer |
| **HLA-DRB1*08:02** | **1** | **15** | **MNFYSKSPKTQSSQH** | **1.1** | **Antigen** | **Non-allergen** | **Negative** | **Non-inducer** | **Inducer** |
| HLA-DRB1*08:02 | 112 | 126 | DNTVHWLLKPSSDHN | 1.6 | Non-antigen | Non-allergen | Negative | Inducer | Non-inducer |
