# Supplementary material for: Structural informatics approach for designing an epitope-based vaccine against the brain-eating Naegleria fowleri
Source: Front Immunol. 2023 Oct 30;14:1284621. doi: 10.3389/fimmu.2023.1284621 (PMC10642955; doi:10.3389/fimmu.2023.1284621)
Supplement: Supplementary file 12 [file Table_5.docx]

**Supplementary Table 5.** Analysis and selection of B-cell epitopes of Hsp70 (The rows in bold show the selected epitopes).

| **Sr No.** | **Start** | **End** | **Peptide** | **Length** | **Antigenicity** | **Allergenicity** | **Toxicity** |
| --- | --- | --- | --- | --- | --- | --- | --- |
| 1. | 75 | 91 | GRKFSDPSVQADMKHWP | 17 | Non-antigen | Non-allergen | Non-toxin |
| 2. | 245 | 259 | KRKHKKDLTENPRAL | 15 | Antigen | Allergen | Non-toxin |
| **3.** | **350** | **363** | **KDFFNGKELCKSIN** | **14** | **Antigen** | **Non-allergen** | **Non-toxin** |
| **4.** | **381** | **403** | **GKETRVLLIDVTPLSLGIETAGG** | **23** | **Antigen** | **Non-allergen** | **Non-toxin** |
| **5.** | **410** | **420** | **ERNTTIPCKKS** | **11** | **Antigen** | **Non-allergen** | **Non-toxin** |
| **6.** | **439** | **450** | **EGERTMTKDNHL** | **12** | **Antigen** | **Non-allergen** | **Non-toxin** |
| **7.** | **497** | **536** | **TITNDKGGLSKEEIEEMLKQAEQMKSQDDELRKEVEAKNH** | **40** | **Antigen** | **Non-allergen** | **Non-toxin** |
| **8.** | **548** | **567** | **VEDPNLAGKISDADKNTIKK** | **20** | **Antigen** | **Non-allergen** | **Non-toxin** |
| 9. | 576 | 592 | IDNNPSASKEDLENKQK | 17 | Non-antigen | Allergen | Non-toxin |
| **10.** | **607** | **655** | **QGAGGAGAGAGGFPGAGAGGFPGAGGFPGAGAGAGGESSTGGAQPKFED** | **49** | **Antigen** | **Non-allergen** | **Non-toxin** |
