# Supplementary material for: Structural informatics approach for designing an epitope-based vaccine against the brain-eating Naegleria fowleri
Source: Front Immunol. 2023 Oct 30;14:1284621. doi: 10.3389/fimmu.2023.1284621 (PMC10642955; doi:10.3389/fimmu.2023.1284621)
Supplement: Supplementary file 13 [file Table_6.docx]

**Supplementary Table 6.** Analysis and selection of B-cell target alleles of Nf23 (The row in bold show the selected epitope).

| **Sr No.** | **Start** | **End** | **Peptide** | **Length** | **Antigenicity** | **Allergenicity** | **Toxicity** |
| --- | --- | --- | --- | --- | --- | --- | --- |
| 1 | 121 | 137 | PSSDHNQLHGPSDVFTR | 17 | Non-antigen | Non-allergen | Non-toxin |
| **2** | **146** | **156** | **PFGGYQEKSGK** | **11** | **Antigen** | **Non-allergen** | **Non-toxin** |
| 3 | 170 | 188 | LHNQQDGRNADGFFSRDPT | 19 | Antigen | Allergen | Non-toxin |
| 4 | 215 | 225 | ASIPLSNGDVF | 11 | Non-antigen | Allergen | Non-toxin |
