# Supplementary material for: Structural informatics approach for designing an epitope-based vaccine against the brain-eating Naegleria fowleri
Source: Front Immunol. 2023 Oct 30;14:1284621. doi: 10.3389/fimmu.2023.1284621 (PMC10642955; doi:10.3389/fimmu.2023.1284621)
Supplement: Supplementary file 14 [file Table_7.docx]

**Supplementary Table 7.** Residues of vaccines involved in the formation of conformational B-cell epitopes.

| **Vaccine construct** | **Residues** | **No. of residues** | **Score** |
| --- | --- | --- | --- |
| V1 | A:G419, A:A420, A:G421, A:A422, A:G423, A:G424, A:E425, A:S426, A:S427, A:T428, A:G429, A:G430, A:A431, A:Q432, A:P433, A:K434 | 16 | 0.881 |
|  | A:S246, A:P247, A:K248, A:T249, A:S251, A:S252, A:Q253, A:H254, A:K256, A:K257, A:D258, A:F259, A:F260, A:N261, A:G262, A:K263, A:E264, A:L265, A:C266 | 19 | 0.735 |
| V2 | A:G343, A:A344, A:G345, A:A346, A:G347, A:G348, A:E349, A:S350, A:S351, A:T352, A:G353, A:G354, A:A355, A:Q356, A:P357, A:K358, A:F359 | 17 | 0.886 |
| V3 | A:G441, A:A442, A:G443, A:A444, A:G445, A:G446, A:E447, A:S448, A:S449, A:T450, A:G451, A:G452, A:A453, A:Q454, A:P455, A:K456 | 16 | 0.88 |
|  | A:S266, A:S268, A:P269, A:K270, A:T271, A:S273, A:S274, A:Q275, A:H276, A:K277, A:K278, A:K279, A:D280, A:F281, A:F282, A:N283, A:G284, A:K285, A:E286, A:L287 | 20 | 0.724 |
