# Supplementary material for: Structural informatics approach for designing an epitope-based vaccine against the brain-eating Naegleria fowleri
Source: Front Immunol. 2023 Oct 30;14:1284621. doi: 10.3389/fimmu.2023.1284621 (PMC10642955; doi:10.3389/fimmu.2023.1284621)
Supplement: Supplementary file 15 [file Table_8.docx]

**Supplementary Table 8.** Molecular interactions between vaccine constructs (V1, V2, and V3) and Toll-like Receptors (TLR2 and TLR4).

| **Vaccine construct** | **Interacting residues** | **Receptor** | **Interacting residues** | **H-bonds** |
| --- | --- | --- | --- | --- |
| V1 | K388, G397, A398, G399, F401, P410, G411, G413, F415, G419, G421, A422, E425 | TLR2 | N294, I304, F325, Y326, S333, E336, S388, N414, K437, Q478, K480, Q316 | 15 |
| V1 | N371, G395, A398, G407, F409, G411, A420, G421, A422, E425, S426, S427, G429. | TLR4 | S184, E266, E270, E321, R322, K324, D325, Q344, I46, N47, V48, N49, E111, N114 | 18 |
| V2 | K299, S301, G323, G324, G331, F333, F339, P340, A346, E349 | TLR2 | I304, F325, Y326, S333, L334, K360, N414, K480, D288, F312 | 12 |
| V2 | G298, K299, G321, A322, G323, G337, G338, G343, G345 | TLR4 | E42, L43, E135, S184, K186, K20, R106, T115 | 10 |
| V3 | K387, A418, G429, F431, F437, R438, A442, E447 | TLR2 | I304, D327, Y332, S333, L334, K360, N414, K480 | 10 |
| V3 | G396, G430, G436, A442, G446. | TLR4 | E27, E42, K186, K20, I46 | 5 |
